# Electrospun Iridium-Based Nanofiber Catalysts for Oxygen Evolution Reaction: Influence of Calcination on Activity–Stability Relation

**DOI:** 10.1021/acsami.4c07831

**Published:** 2024-09-18

**Authors:** Miklós Márton Kovács, Birk Fritsch, Leopold Lahn, Julien Bachmann, Olga Kasian, Karl J. J. Mayrhofer, Andreas Hutzler, Dominik Dworschak

**Affiliations:** † Forschungszentrum Jülich GmbH, Helmholtz Institute Erlangen-Nürnberg for Renewable Energy (IET-2), 91058 Erlangen, Germany; ‡ Friedrich-Alexander-Universität Erlangen-Nürnberg, Department of Chemical and Biological Engineering, 91058 Erlangen, Germany; ¶ Helmholtz-Zentrum Berlin für Materialien und Energie GmbH, Dynamic Electrocatalytic Interfaces, Hahn-Meitner-Platz 1, 14109 Berlin, Germany; § Friedrich-Alexander-Universität Erlangen-Nürnberg, Department of Materials Science and Engineering, 91058 Erlangen, Germany; ∥ Friedrich-Alexander-Universität Erlangen-Nürnberg, Chemistry of Thin Film Materials, IZNF, 91058 Erlangen, Germany

**Keywords:** Acidic water electrolysis, Enhanced catalyst utilization, Electrospinning, Temperature-controlled synthesis, Catalyst morphology, Iridium oxide

## Abstract

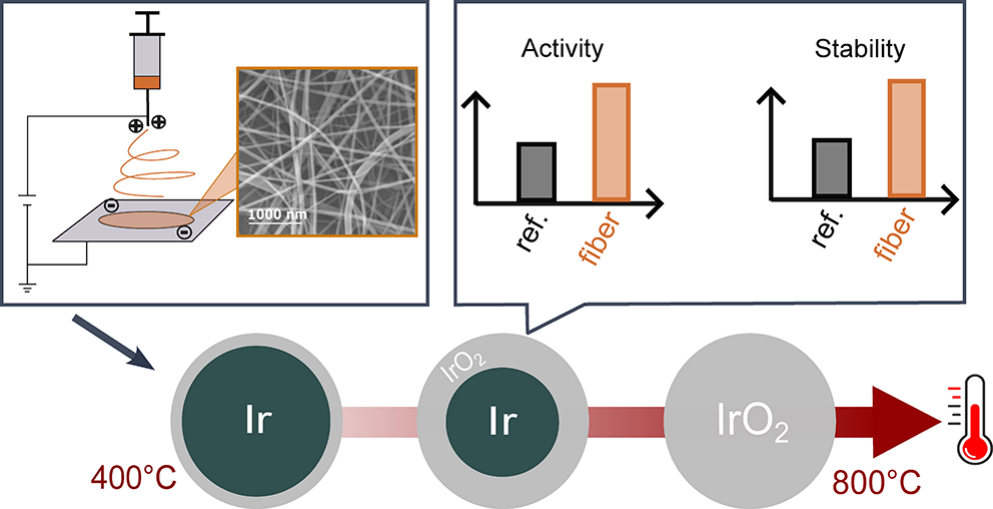

The enhanced utilization of noble metal catalysts through highly porous nanostructures is crucial to advancing the commercialization prospects of proton exchange membrane water electrolysis (PEMWE). In this study, hierarchically structured IrO_*x*_-based nanofiber catalyst materials for acidic water electrolysis are synthesized by electrospinning, a process known for its scalability and ease of operation. A calcination study at various temperatures from 400 to 800 °C is employed to find the best candidates for both electrocatalytic activity and stability. Morphology, structure, phase, and chemical composition are investigated using a scale-bridging approach by SEM, TEM, XRD, and XPS to shed light on the structure–function relationship of the thermally prepared nanofibers. Activity and stability are monitored by a scanning flow cell (SFC) coupled with an inductively coupled plasma mass spectrometer (ICP-MS). We evaluate the dissolution of all metals potentially incorporated into the final catalyst material throughout the synthesis pathway. Despite the opposite trend of performance and stability, the present study demonstrates that an optimum between these two aspects can be achieved at 600 °C, exhibiting values that are 1.4 and 2.4 times higher than those of the commercial reference material, respectively. The dissolution of metal contaminations such as Ni, Fe, and Cr remains minimal, exhibiting no correlation with the steps of the electrochemical protocol applied, thus exerting a negligible influence on the stability of the nanofibrous catalyst materials. This work demonstrates the scalability of electrospinning to produce nanofibers with enhanced catalyst utilization and their testing by SFC-ICP-MS. Moreover, it illustrates the influence of calcination temperature on the structure and chemical composition of the nanofibers, resulting in outstanding electrocatalytic performance and stability compared to commercial catalyst materials for PEMWE.

## Introduction

The need for electricity conversion and energy storage from sustainable resources has fostered a climate of innovation, prompting diverse approaches to address the requirements of the energy transition. Recently, water electrolysis has become a key approach to converting excess electrical energy into hydrogen, although the reaction itself has been known for centuries. Proton exchange membrane water electrolysis (PEMWE) has attracted a lot of attention lately and is considered a promising technology for industrial-scale hydrogen production. Despite the use of scarce and expensive noble metals, PEMWE technology has been thoroughly investigated worldwide, as it can be operated with renewable energy resources of an intermittent nature.^1^ In the electrodes of a single electrolyzer cell, the feedstocks of the catalyst materials are platinum for the cathodic hydrogen evolution reaction (HER) and iridium for the anodic oxygen evolution reaction (OER).

OER poses the main obstacle to the widespread deployment of PEMWE due to its low intrinsic reaction rate and harsh environment. To overcome this, a loading of 1–2 g_Ir_ cm^–2^ per cell is required. This has prompted concerted efforts to decrease the amount of Ir applied.^2−4^ The amount of Ir used is perceived as too high to satisfy the criteria of a global energy transition.^5,6^ Recent techno-economic studies reveal a need to reduce Ir loading substantially to meet the requirements of water electrolysis in the gigawatt scale.^7,8^ The two basic factors that make PEMWE technology more affordable are enhanced kinetics and the low loading of noble metals. Iridium oxide (IrO_*x*_) is considered to be a state-of-the-art material for OER in acidic water electrolysis, creating a compromise between electrocatalytic activity to OER and stability during cell operation.^9,10^ Although Ir as a pure metal is also used as a benchmark catalyst material for OER, the actual catalyst material remains IrO_*x*_, since the high anodic potentials required for OER inevitably lead to the oxidation of the electrode material.^11,12^

Multiple structural chemical formulas have been investigated due to diverse oxidation states of Ir, including amorphous and crystalline IrO_*x*_.^13−15^ Previous *operando* X-ray absorption spectroscopy (XAS) and X-ray photoelectron spectroscopy (XPS) experiments have suggested that the activation of amorphous IrO_*x*_ predominantly takes place within the deeper layers of the material’s surface, leading to a lower energetic barrier for the OER through an electronic doping effect.^16,17^ A high number of defects, the presence of Ir^III^OOH groups, and the porosity in the hydrous IrO_*x*_ thus ensure that more lattice oxygen atoms are available for OER, resulting in higher OER activity.^18^ Nevertheless, amorphous IrO_*x*_ exhibits a major drawback in terms of durability compared with its crystalline counterpart.^19,20^ Iridium oxide with an ordered rutile structure is one of the most stable catalysts for the OER in terms of dissolution. In fact, calcination at elevated temperatures enables, alongside sintering, the transition of amorphous structures to more crystalline structures. However, this entails a decrease in the catalyst surface area, thus reducing OER activity.^15,21,22^ Multiple calcination studies have been proposed to benchmark the electrocatalytic activity and stability of IrO_*x*_-based catalyst materials.^23,24^ Cherevko et al. found that there was not necessarily a trade-off between activity and stability for IrO_*x*_ films on a Ti substrate in the calcination range of 250–550 °C.

The modification of the catalyst nanostructure is one of the approaches used to mitigate performance losses and improve the durability of low-loaded anodes. Several morphologies have been reported to serve as high-performance IrO_*x*_-based catalysts including nanoparticles,^24−27^ nanoflowers,^28,29^ or dendrites.^30^ The most promising approaches to catalyst design involve augmenting the aspect ratio, determining the relationship between the shortest and longest dimensions of the catalyst particles.^31−33^ Particles possessing the highest aspect ratios are commonly classified as one-dimensional (1D) nanostructures, or simply referred to as nanofibers (NFs), which have attracted increasing attention in catalyst research efforts related to acidic water electrolysis.^34−38^ If the fiber diameter is below 100 nm, electron confinement occurs, yielding high electric conductivity which is accompanied by high surface energy and high catalytic surface activity.^39^

Electrospinning is an easy and reliable way of generating nanofibers.^40−44^ Moreover, as a synthesis tool in fuel cell and electrolysis applications, it has advantages such as being easy to handle, scalable, and versatile in terms of composition and morphology.^35,45,46^ Due to their unique nanofibrous heterostructure, which gives them a large specific surface area and high porosity, the synthesized IrO_*x*_ NFs exhibit improved OER properties in acidic media assuring enhanced ion transport.^47,48^ However, high porosity, which indicates a larger surface area, often corresponds to a higher dissolution rate in electrochemical reactions.^17−20^ A significant scientific challenge is, therefore, to enhance intrinsic stability while also promoting the formation of an extended surface structure.

In this study, we synthesized IrO_*x*_-based NFs at various calcination temperatures by means of electrospinning. The electrocatalytic activity and stability of IrO_*x*_ NFs are compared in an acidic electrolyte (0.1 M HClO_4_) with IrO_*x*_ nanoparticles (NPs) from Alfa Aesar as the reference material. Changes of the crystal lattice across macro- and microscales and the electronic structure during calcination are investigated by X-ray diffraction (XRD), selected area electron diffraction (SAED), and X-ray photoelectron spectroscopy (XPS), respectively. Geometry and morphological stability are studied by high-angle annular dark-field scanning transmission electron microscopy (HAADF-STEM) and high-resolution transmission electron microscopy (HR-TEM). The dispersion of iridium and oxygen within NFs is observed by scanning transmission electron microscopy coupled with energy-dispersive X-ray spectroscopy (STEM-EDXS). Electrocatalytic activity toward the OER as well as the dissolution rate are recorded by an *operando* scanning flow cell coupled to an inductively coupled plasma mass spectrometer (SFC-ICP-MS). The dissolution rate is quantified using the stability number (S) as a key figure of merit.^20^

This study provides a thorough analysis of the effect of the calcination temperature on the structure and chemical composition of the IrO_*x*_ NFs as well as their function in the electrochemical OER in acidic water electrolysis in a dynamic real-time assessment.

## Results and Discussion

### Physical Characterization

**Figure 1:**
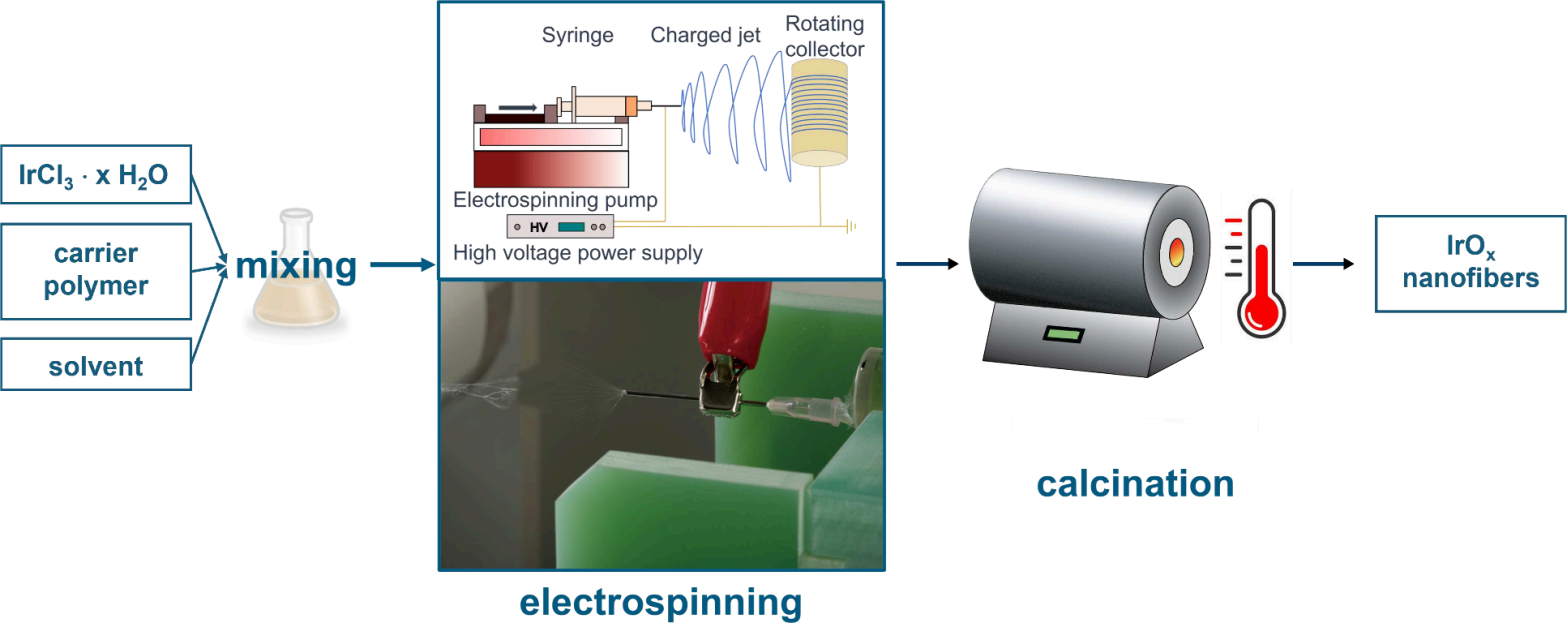
Complete workflow of the synthesis of 1D IrO_*x*_ nanofibers. Calcination takes place in a tube furnace for *t* = 2 h at temperatures of *T* = 400 °C, 500 °C, 600 °C, 700 °C, and 800 °C. Photo of the NEU Nanofiber Electrospinning Unit is adapted with permission from the producer Kato Tech Co.

To increase the accessibility of catalyst active centers consisting of iridium oxide, high-aspect-ratio nanofibers are electrospun and calcined at various temperatures. The complete workflow for the nanofiber synthesis process can be found in Figure 1. The morphology and thickness of the nanofibers are determined before and after calcination by scanning electron microscopy (SEM). In Figure S1.a, SEM images of each catalyst material are depicted before and after calcination. Mean values of fiber diameters are summarized in Figure S1.b. Before calcination, the fiber thickness varies significantly from 88 ± 23 nm to approximately 413 ± 125 nm, which might be a result of the polymer content of the precursor solution.

During calcination, we observe a considerable decrease in the nanofiber diameter and its standard deviation due to the removal of chloride from the precursor salt IrCl_3_ and the oxidation of PVP into gaseous products, for example CO_2_. After calcination, the nanofibers exhibit a similar thickness (from 44 ± 8 nm to 57 ± 9 nm). The negligible increase in the fiber diameter is attributed to the oxidation of iridium depending on the final temperature and the duration of calcination. A preliminary elemental study was also conducted by SEM-EDXS at least three times for every sample calcined at 400 °C, 500 °C, 600 °C, 700 °C, and 800 °C (Figure S1.c for the example of IrO_*x*_ 600) to ensure that Cl had been substantially removed.

To gain an understanding about the individual steps of temperature-dependent mass loss, thermogravimetric analysis (TGA) is carried out with the samples before and after calcination. Due to calcination under a synthetic air atmosphere, the nanofibers containing IrCl_3_ and PVP transform to iridium oxide (Figure S2). As for the samples before calcination, chemisorbed water is released in the first step at around 100 °C. Second, the carrier polymer PVP is entirely removed at its decomposition temperature of 350 °C, as was reported in the literature.^49^ Beyond this temperature, we observe no notable decrease in mass for both noncalcined and calcined samples. In fact, they exhibit a slight gain in mass at high temperatures due to further oxidation except for the IrO_*x*_ sample calcined at 800 °C, indicating that the nanofibers are fully oxidized (IrO_2_). Above 900 °C, the decomposition of IrO_*x*_ to metallic Ir may result in a mass loss of about 5.6%. Based on our observations, a lower calcination temperature leads to a greater mass gain, primarily as a result of further oxidation.

**Figure 2:**
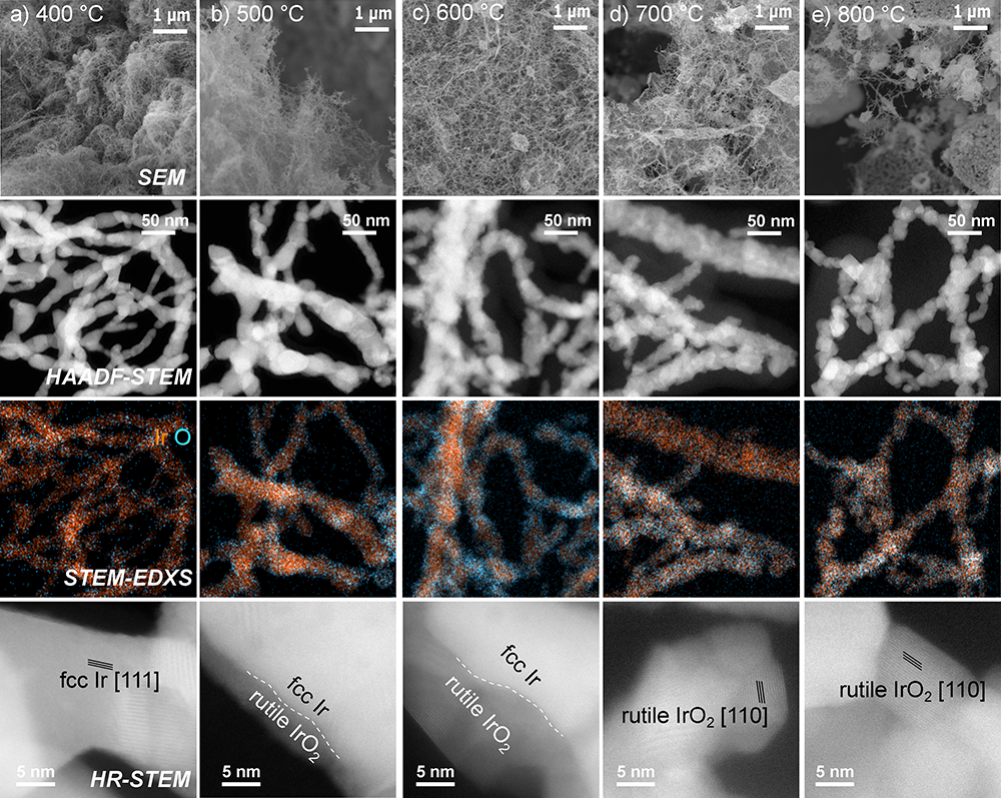
Influence of calcination on the composition of IrO_*x*_ NFs at *T* = (a) 400 °C, (b) 500 °C, (c) 600 °C, (d) 700 °C, and (e) 800 °C: SEM, HAADF-STEM and STEM-EDX spectrum images of the Ir (orange) and O (blue) content. HR-STEM shows the degree of oxidation throughout the nanofibers.

**Figure 3:**
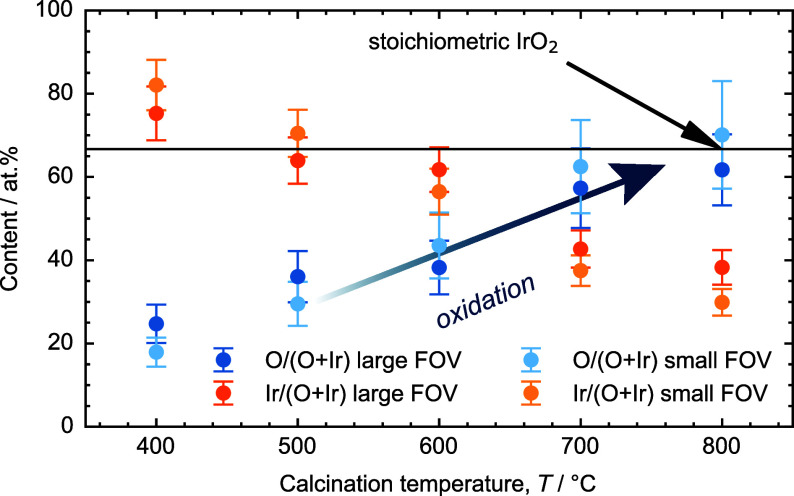
Atomic proportion of O (blue) and Ir (orange) calculated in relation to the overall composition in the temperature range of 400 to 800 °C in a large and a small field of view (FOV) of (290.3 nm)^2^ and (580.7 nm)^2^, respectively. The standard deviation is determined in three FOVs within a sample.

**Figure 4:**
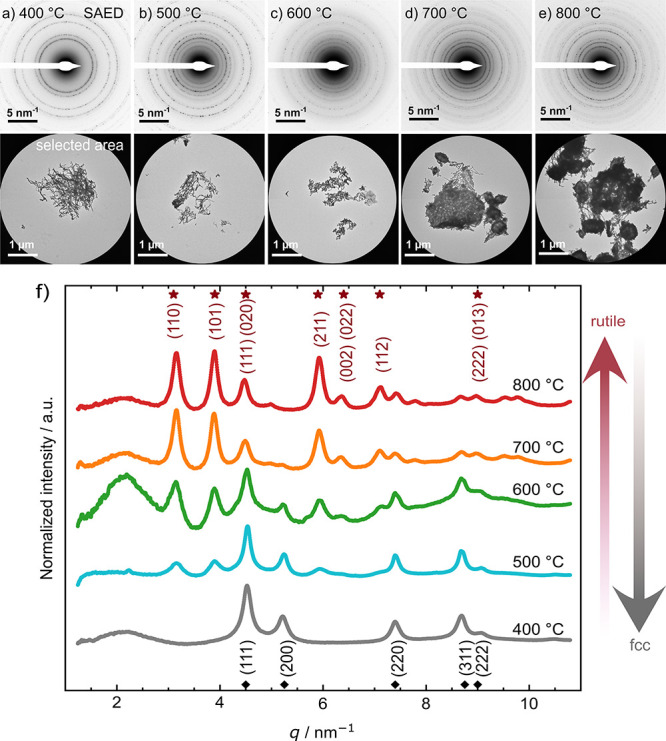
SAED patterns and the selected areas of IrO_*x*_ nanofibers calcined at (a) 400 °C, (b) 500 °C, (c) 600 °C, (d) 700 °C, and (e) 800 °C to determine the crystallinity of the samples. (f) The effect of calcination on structure: Normalized intensity vs reciprocal lattice vector *q* (nm^–1^) of SAED patterns of rutile IrO_2_ (asterisk) and fcc Ir metal (diamond) morphologies in IrO_*x*_ nanofibers. *q* is the reciprocal lattice vector where 1/*q* corresponds to lattice plane distances in real space.

The physicochemical characterization of IrO_*x*_ based nanofibers involves *ex situ* HAADF-STEM, HR-STEM coupled with EDXS (Figures 2, 3 and S3), and SAED (Figure 4) experiments to determine morphology, crystallinity, and the oxidation grade of the nanofibers at each calcination temperature. In Figure 2, we add SEM images for comparison, which — alongside HAADF-STEM — demonstrate that the fibrous mat structure is preserved after the calcination process. Nevertheless, the fibers undergo sintering at temperatures of 700 and 800 °C, resulting in the decrease of the specific surface area.

In order to unfold the chemical composition of the nanofibers, elemental mapping is applied by STEM-EDXS. In Figure 3, the atomic proportions of oxygen (blue) and iridium (orange) are depicted, as calculated from the STEM-EDXS measurements. The lowest O:Ir ratio (approximately 1:4) is observed in IrO_*x*_ calcined at 400 °C. HR-STEM sheds light on the predominant presence of face-centered cubic (fcc) Ir at 400 °C throughout a single nanofiber, as confirmed by SAED analysis below (Figure 4). At 500 and 600 °C, a core–shell nanofiber structure is identified in which the core comprises fcc Ir, while overlapping fcc Ir 111 and rutile IrO_2_ 111/020 reflections can be observed in the shell. This phenomenon results in an increase in the overall O:Ir ratio (approximately 1:2 and 2:3, respectively). Over the course of the calcination study, the proportion of incorporated oxygen increases, and rutile IrO_2_ becomes prevalent at 700 °C (O:Ir = 3:2). The stoichiometric composition of IrO_2_ (O:Ir = 2:1, Figure 3) is attained at 800 °C, which is in good agreement with TGA results (Figure S2). As for chlorine content of the precursor salt IrCl_3_, STEM-EDXS reveals that 1–2 at. % of residual Cl can be found in all nanofibrous samples which is in accordance with SEM-EDX results of Figure S1.c.

In Figure 4, SAED results confirm the crystalline nature of all samples throughout the calcination study. According to the lattice spacing, the diffraction pattern of IrO_*x*_ calcined at 400 °C is found to correspond to the overlapping of predominant fcc Ir 111 (metallic Ir) and a small amount of rutile IrO_2_ 111/020. By increasing the calcination temperature, additional diffraction rings appear that correspond to rutile IrO_2_. Analyzing the distortion-corrected radial profile (Figure S5) reveals that the intensities of the rutile peaks gradually increase at the expense of the fcc signal. Nonetheless, we note that all calcined specimens contain fcc grains, as depicted in Figure 4f.

**Figure 5:**
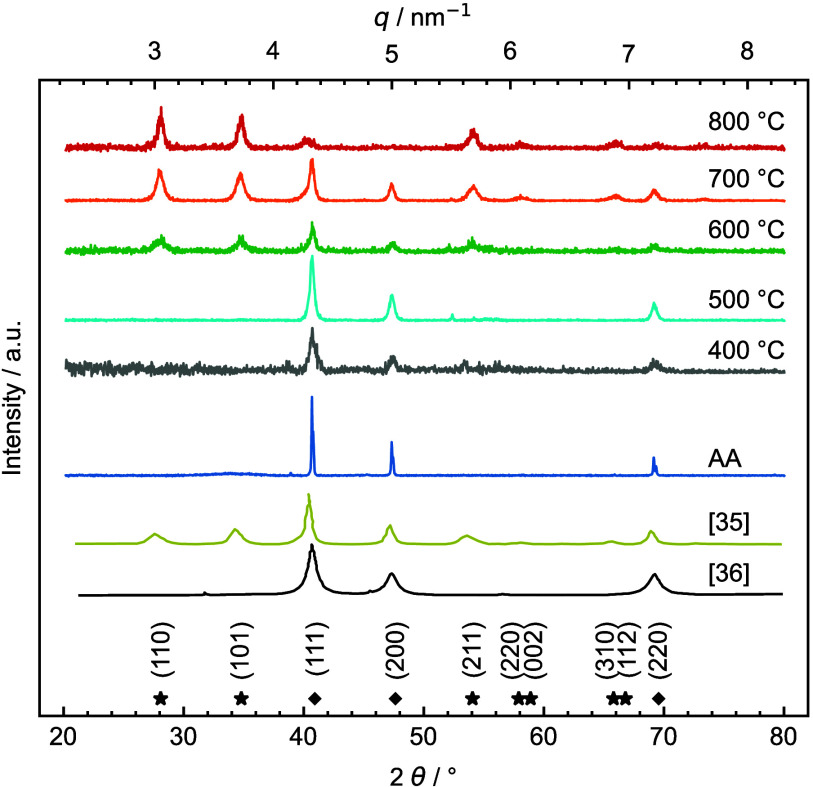
X-ray diffraction patterns of the IrO_*x*_ and IrO_2_ nanofibers (NF) as well as commercial IrO_*x*_ Alfa Aesar nanoparticles (IrO_*x*_ AA). Characteristic locations of the Bragg diffraction of fcc Ir (diamond) and rutile IrO_2_ (asterisk) are indicated at the bottom.^51^ Diffraction patterns of previous reports are reproduced and adapted with permission from refs (35 and 36).

To establish scale-bridging between SAED and X-ray diffraction (XRD), profiles from the latter technique are acquired to comprehensively understand the crystallinity of the IrO_*x*_-based nanofibers (Figure 5). The peaks at 40.8°, 47.4°, and 69° are attributed to the 111, 200, and 220 reflection from the metallic iridium phase,^50^ which is predominant in the reference material IrO_*x*_ AA as well as IrO_*x*_ calcined at 400 and 500 °C. The diffraction pattern of IrO_*x*_ AA nanoparticles is in accordance with the work of Moon et al.^35^ and Hegge et al.^36^ Crystalline morphologies of each diffraction pattern are determined in accordance with the previous study of Pfeifer et al.^51^ By increasing the calcination temperature, the reflection peaks 110, 101, and 211 of rutile IrO_2_ appear and reach a maximum IrO_2_/Ir ratio at 800 °C. At the same time, the intensity of metallic iridium decreases substantially. This observation corroborates the results of STEM-EDXS and HR-STEM depicted in Figures 2 and 3.

**Figure 6:**
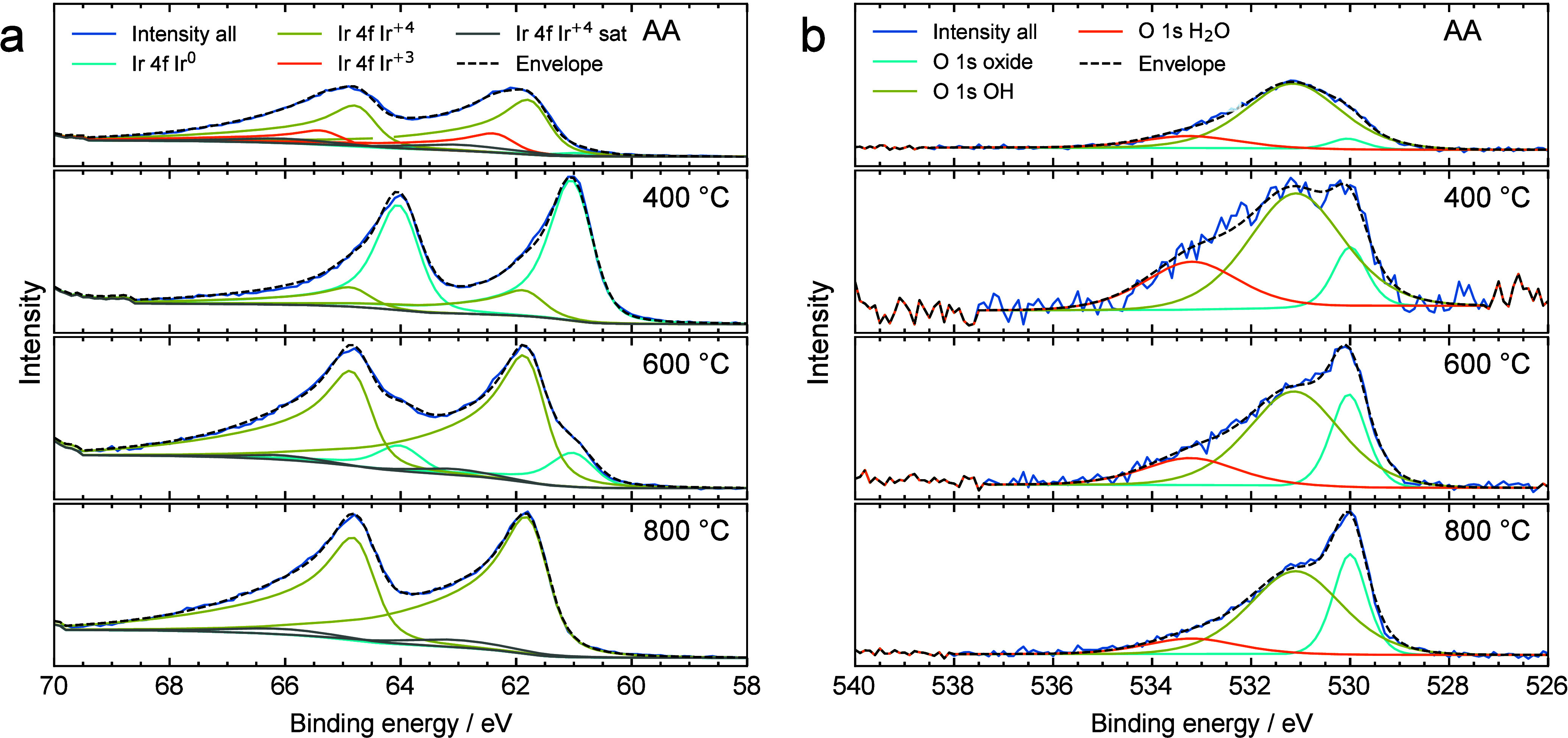
XPS analysis (a) Ir 4f core-level spectra and (b) O 1s core-level spectra of IrO_*x*_ nanofibers calcined at *T* = 400 °C, 600 °C, and 800 °C as well as the commercial reference material. C 1s and survey spectra of the samples can be found in Figure S6.

To determine the chemical state of the topmost surface layers and validate STEM-EDXS results by scale-bridging, *ex situ* XPS measurements of particular samples (IrO_*x*_ 400, 600, and 800 as well as the reference material IrO_*x*_ AA) are performed. Figure 6a shows the narrow scan of the Ir 4f level, featuring a peak with splitting to Ir 4f_7/2_ and Ir 4f_5/2_.^52,53^ The XP spectra of the benchmark IrO_*x*_ AA feature two main contributions at 61.7 and 62.3 eV, which correspond to Ir^+4^ and Ir^+3^, respectively. Moreover, a small contribution from metallic Ir is identified at 60.9 eV. This data are in line with numerous literature reports confirming that the Alfa Aesar catalyst is a mixture of crystalline rutile and amorphous hydrous oxides of Ir.^51,52,54^

The deconvolution of the Ir 4f spectra for IrO_*x*_ calcined at 400 °C comprises two components. The first component, observed at 61.0 eV, can be assigned to metallic Ir (Ir^0^); the second one at 61.8 eV corresponds to Ir^+4^ in an oxide lattice, for example IrO_2_.^53,55^ These results indicate incomplete oxidation during calcination at 400 °C. According to the XPS data, the ratio between oxidized and metallic Ir is 0.3 (Table S3), which is in good agreement with the STEM-EDXS results. With increasing calcination temperature up to 600 °C, there is no observable shift in peak positions. Nevertheless, the content of Ir^0^ diminishes to 15.3 at. %, and the ratio between oxidized and metallic Ir rises to 5.5. The Ir 4f spectrum of the catalyst calcined at 800 °C features a single contribution, which corresponds to the Ir^+4^ oxidation state in IrO_2_. In contrast to the benchmark catalyst IrO_*x*_ AA, we observe no traces of hydrated Ir (Ir^+3^ in the calcined samples), which is likely due to the precise temperature control during the calcination program. This approach resulted in enhanced stability of the nanofibrous materials.

The O 1s spectra feature three main contributions, corresponding to lattice oxygen (530.0 eV), oxygen in the OH groups (531.1 eV), and water molecules (533.2 eV). The broad O 1s peaks (Figure 6b) reveal substantial contributions from the hydroxyl groups. With increasing temperature, the contribution from lattice oxygen increases and ultimately dominates the shape of the O 1s peak measured on the sample calcined at 800 °C. This result indicates the presence of lattice oxygen on the surface. In all spectra, we observe the contribution of hydroxyl groups which rather result from the exposure to atmospheric conditions during the measurement and not from a replacement by lattice oxygen.^19,56,57^ An additional contribution to the O 1s peak is at 533.2 eV, which is attributed to adsorbed water, albeit with a slightly decreasing ratio with increasing temperature. This peak can originate from the sample preparation for XPS in a mixed aqueous medium and from air exposure.

### Electrochemical Characterization

The electrocatalytic activity toward the OER is characterized by the electrochemically active surface area (*ECSA*), which is determined by cyclic voltammetry (CV) measurements of the drop-cast catalyst spots. Hereby, we calculate the *ECSA* by taking the average of the cathodic and the anodic charge of IrO_*x*_ thin films with CV data between 0.4 and 1.3 V vs RHE as suggested in the literature.^11,58^ As for the specific charge, 596 (±21) μC cm^–2^ is applied for IrO_*x*_.^59^ We experienced the largest *ECSA* for IrO_*x*_ AA nanoparticles, which is the result of the porous mixed oxide structure of the surface (Figure 6). IrO_*x*_ calcined at 600 °C exhibit the largest *ECSA* value among the nanofibrous catalysts, whereas the other four materials show similarly large active surface areas. The summarized data with the respective CV curves can be found in Figure S7a and in Table S5.

A larger surface area of the reference material is also observed in BET measurements compared to the selected IrO_*x*_ nanofibers (calcined at 400 °C, 600 °C, and 800 °C); however, the predominantly metallic Ir nanofibers calcined at 400 °C outperform IrO_*x*_ AA and has the largest BET surface area (29.81 m^2^ g^–1^_Ir_ and 33.57 m^2^ g^–1^_Ir_, respectively). The difference between CV and BET results of active surface area could stem from the influence of the calcination temperature on the pseudocapacitance.^23^

**Figure 7:**
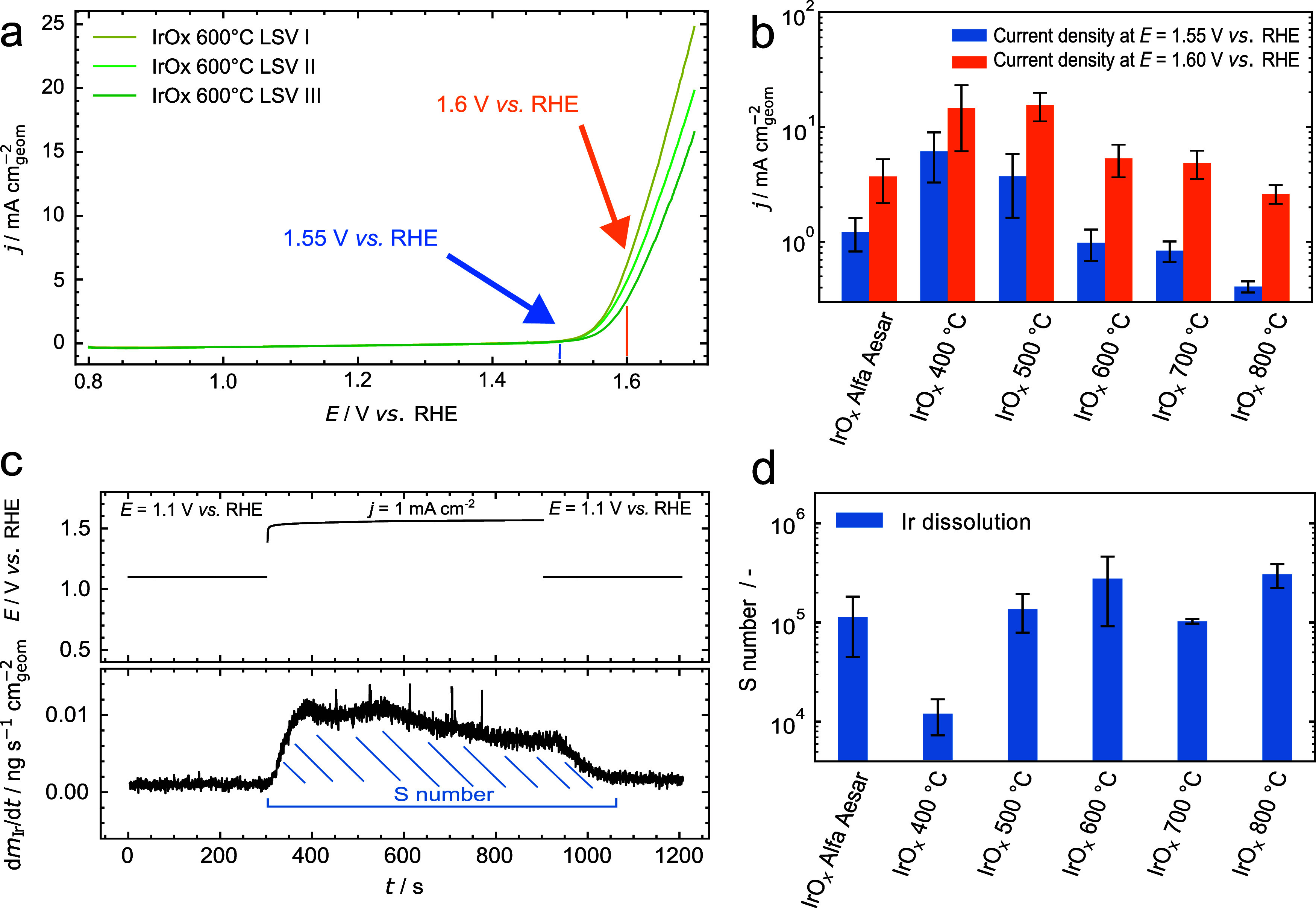
(a) Linear sweep voltammetry (*iR* corrected) in the entire potential region of 0.80 to 1.70 V vs RHE in 0.1 M HClO_4_ for the example of IrO_*x*_ nanofibers calcined at 600 °C. (b) OER activity for all IrO_*x*_ nanofibers and the reference material at 1.55 and 1.60 V vs RHE. Scan rate was 20 mV s^–1^. The data are based on at least three measurements of each specimen. (c) Definition of the stability number S determined by dissolution measurements during a galvanostatic hold at *j* = 1 mA cm^–2^ of IrO_*x*_ nanofibers after calcination at *T* = 400 °C, 500 °C, 600 °C, 700 °C, and 800 °C and the benchmark catalyst in 0.1 M HClO_4_. The experiments were conducted in an SFC-ICP-MS setup. (d) Dissolution of Ir quantified with the stability number S for all IrO_*x*_-based catalyst materials investigated. The stability number is calculated from the ratio of oxygen evolved and iridium dissolved.^20^

To shed light on the structure–function relationship of the nanofibers with their electrochemical performance and durability, polarization curves of IrO_*x*_ specimens are recorded by linear sweep voltammetry (LSV) to determine the electrocatalytic activity at both 1.55 and 1.60 V vs RHE, as depicted in Figure 7a with the example of IrO_*x*_ calcined at 600 °C in three distinct measurements. LSVs are recorded up to 1.70 V vs RHE to avoid intense macroscopic bubble accumulation on the catalyst surface and artifacts in higher dissolution rates.^60^ The results are normalized to the geometric surface area of each catalyst spot and are summarized for all specimens examined in Figure 7b. We also provide a short overview of LSVs, which are recorded after sample conditioning by cyclic voltammetry and compared to the results obtained without conditioning (CVs in Figure S7.a; a summary can be found in Table S4).

At 1.55 V vs RHE, the IrO_*x*_ nanofibers calcined at 400 and 500 °C exhibit similar OER activity (6 ± 3 and 4 ± 2 mA cm^–2^, respectively). Both specimens significantly surpass the electrocatalytic activity of the reference material (1.2 ± 0.4 mA cm^–2^), whereas the current densities of IrO_*x*_ NFs calcined at 400 and 500 °C are in the same range at 1.60 V vs RHE ((1.5 ± 0.9)·10^1^ mA cm^–2^ and (1.6 ± 0.4)·10^1^ mA cm^–2^, respectively, compared to 4 ± 2 mA cm^–2^ of IrO_*x*_ AA). OER activity for all materials can be found in Table S4. This might be the result of the larger specific surface area of nanofibers and their chemical composition, as discussed in the STEM-EDXS records of Figures 2 and 3.^13,58,61,62^ Other factors might include the relative uncertainty of the catalyst loading in the spots and the passivation of the GC backing electrode in the OER region.^63^

The incorporation of oxygen into the nanofibers creates a core–shell structure already at 500 °C (core: metallic Ir, shell: IrO_2_), resulting in an enlarged number of defects in the topmost surface layers, i.e. higher porosity. Despite the observation of no hydrous IrO_*x*_ in the nanofibrous specimens (Figure 6a), this mixed porous structure of polycrystalline Ir and IrO_*x*_ exhibits higher electrocatalytic activity than the reference material. Mass activity (*MA*) results also support this trend of the OER activity among the catalyst materials investigated. The values of the specific activity (*SA*) are selected for the samples IrO_*x*_ AA and the NFs calcined at 400 °C, 600 °C, and 800 °C. By means of BET measurements, we calculated the highest *SA* for IrO_*x*_ NFs calcined at 400 °C (4.36 mA cm^–2^_BET_ which appears to be almost twice as large as the *SA* of the complete rutile IrO_2_ nanofibers (calcined at 800 °C). All three nanofibrous materials outperform the reference of Alfa Aesar nanoparticles at *E* = 1.60 V vs RHE. The results are summarized in Table S5.

An established core–shell structure is obtained at 600 °C, in which the shell thickness of rutile IrO_2_ is estimated to be approximately 10 nm (HR-STEM record in Figure 2). This results in a marked reduction of the OER activity in comparison with the IrO_*x*_ samples calcined at 400 and 500 °C, accounting for 0.98 mA cm^–2^ and 5.33 mA cm^–2^ at 1.55 and 1.60 V vs RHE, respectively. The Ir core fully oxidizes at elevated calcination temperatures achieving the stoichiometric composition of rutile IrO_2_ at 800 °C, leading to a further decline in the OER activity by 55% and 49% compared to IrO_*x*_ calcined at 600 and 700 °C, respectively. Besides, we take electrochemical control measurements with IrO_*x*_ calcined at 600 °C for 10 h to obtain evidence that the morphology — and with that, the electrochemical behavior, is not dependent on the calcination time but on the temperature applied. Hereby, we obtain CV curves similar to IrO_*x*_ calcined at 600 °C for 2 h and we can not observe an electrochemical character of the complete rutile IrO_2_. Therefore, a five times longer calcination time does not appear to be sufficient to achieve the same morphology as at 800 °C for 2 h.^51,64^

As depicted in Figure 2 (STEM-EDXS and HR-STEM) and summarized in Figure 3, the ratio of O:Ir throughout a single nanofiber increases; i.e., fcc Ir sites are oxidized to rutile IrO_2_ to a larger extent. We find a similar explanation when the oxidation process is investigated by *ex situ* XPS on thin films of IrO_*x*_ NFs (Figure 6). In this scale-bridging approach, we observe a decrease in the ratio of Ir^0^/Ir^+4^ when the calcination temperature is set higher. We thus obtain the same outcome of Ir^+4^ accounting for 100 at. % of the Ir content, confirming the rutile structure of IrO_*x*_ calcined at 800 °C. For more details on the fiber compositions, please refer to Table S3.

However, we experience an inverse trend, as we gain insight into the relationship between calcination temperature and electrochemical stability. We insert a current-controlled step into the characterization protocol at 1 mA cm^–2^ for *t* = 600 s in order to model the OER. The dissolution is quantified by determining the stability number S.^20^ This figure of merit quantifies the direct relationship between the amount of oxygen evolved and the quantity of metallic iridium dissolved during the OER (Figure 7c). IrO_*x*_ calcined at 800 °C exhibits the highest intrinsic stability as depicted in Figure 7d. It is more than 1 order of magnitude higher than IrO_*x*_ calcined at 400 °C and approximately three times higher than the reference material. In previous studies, the outstanding stability of IrO_2_ calcined at higher temperatures was related to low porosity and the lack of oxygen vacancies of absent amorphous IrO_*x*_.^20,23^

Nevertheless, the modest stability of IrO_*x*_ calcined at 400 °C can be ascribed to the subsurface deprotonation, lowering the barrier for the dissolution of Ir species. Together with the lower oxygen connectivity for less crystalline oxides, this might explain the correlation between crystallinity and stability. As for IrO_*x*_ calcined at 600 °C, we obtain a balance of good electrocatalytic activity and enhanced stability compared to the reference IrO_*x*_ AA. We believe that the morphology of the core–shell nanofibers (shown in Figure 2) has an impact on the electrocatalytic behavior of the sample, namely the improved stability number of IrO_*x*_ 600 compared to IrO_*x*_ 700. The oxidized outer layer of Ir may act as a passivation layer, protecting the metallic Ir core from extended dissolution but still exhibiting high activity toward OER.^17^ Overall, we observe an inverse correlation between electrocatalytic activity and stability of the IrO_*x*_ nanofibrous catalyst materials highlighting the impact of the structure and chemical composition of the surface on these properties.^9,65^

**Figure 8:**
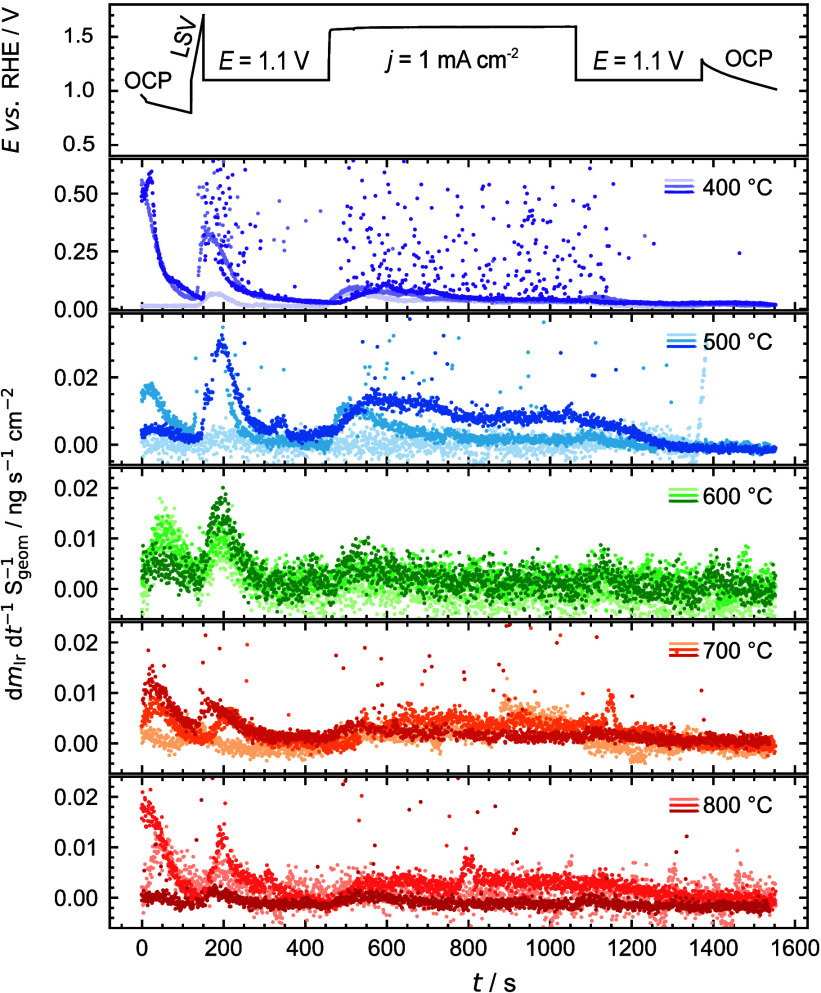
Overall electrochemical characterization protocol (top axis) as well as the dissolution rates of Ir in the IrO_*x*_ nanofibers calcined at 400 °C, 500 °C, 600 °C, 700 °C, and 800 °C. Three separate catalyst spots are analyzed at each temperature as depicted by color shading. The experiments are conducted by an SFC instrument coupled to ICP-MS in 0.1 M HClO_4_. The characterization protocol consisted of 1. OCP, 2. LSV from *E* = 0.80 or 1.10 V to 1.70 V, 3. potentiostatic hold at *E* = 1.10 V, 4. galvanostatic hold at *j* = 1 mA cm^–2^, 5. potentiostatic hold at *E* = 1.10 V, 6. OCP, and 7. EIS was used to determine the real component of the impedance stemming from the electrolyte. All potentials are defined vs RHE.

Besides the dissolution of Ir, we also monitor the dissolved amounts of Ni, Cr, Fe, and Al to ensure that these elements do not contaminate the final catalyst material throughout the synthesis route. In the first step, a needle made of a stainless steel alloy consisting of Ni, Cr, and Fe, is used to produce nanofibers. The dissolution of these metals in stainless steel has been reported in an SFC-ICP-MS setup for the near-neutral pH range.^66^ Moreover, the fibers are collected on an Al foil and calcined in an alumina combustion boat (Coors Al_2_O_3_). The real-time dissolution profiles of Ir are depicted in Figure 8 for all calcined specimens. The dissolution profiles of all metals can be found in Figure S8 with the example of IrO_*x*_ calcined at 600 °C as well as the other metals investigated in Figures S9–S12. The electrochemical protocol is added at the top of each figure, where the dissolution of those species is predominantly due to the establishment of contact between the materials and the flowing electrolyte through the electrochemical cell.

Furthermore, dissolution peaks can be observed in IrO_*x*_ calcined at 400 and 500 °C at the beginning of the galvanostatic step at 1 mA cm^–2^ due to a rapid increase in the potential from 1.10 V up to the OER region of 1.50–1.60 V vs RHE (the exact values for each specimen are summarized in Figure S7.b). The degree of this rapid increase in the dissolution rates noticeably declines when the calcination temperature is set higher. As for the accompanying metals Ni, Cr, Fe, and Al, however, we experience no dissolution over the entire galvanostatic hold, questioning the electrochemical nature of those peaks. Still, the influence of these metals on the iridium dissolution needs to be further investigated.

## Conclusion

In summary, an unconventional synthesis route is demonstrated with the comprehensive characterization of hierarchically structured IrO_*x*_ nanofibers. We apply a scalable method for the production of nanofiber-based catalyst materials for acidic water electrolysis. Through the combination of electrospinning and calcination in a following step, we create catalyst materials of 1D geometry for OER, which fully expose the active sites on the catalyst surface. We show that the nanofibers remain unaffected during the calcination study of 400 to 800 °C in terms of morphology, although the fiber diameter remains constant at about 0.06 ± 0.01 μm. Using a scale-bridging approach, we shed light on the oxidation mechanism of the nanofibers throughout the calcination study and determined their chemical composition by STEM-EDXS, as confirmed by XPS data. We applied distortion-corrected SAED to reveal the relationship between the structure (crystallinity) and function (electrocatalytic activity and stability) of a single nanofiber. We confirm this relationship by X-ray diffraction patterns and thorough electrocatalytic characterization. We demonstrate the improved OER activity and stability of IrO_*x*_-based nanofibers during OER compared to our benchmark material measured by the SFC-ICP-MS setup. Specific activity values were found to exceed that of the reference material for all nanofibrous samples investigated at 1.60 V vs RHE. Indeed, all nanofibers calcined at *T* > 400 °C outperformed the reference IrO_*x*_ AA in terms of stability (S numbers are larger by 31%, 137%, 13%, and 360% for IrO_*x*_ calcined at 500 °C, 600 °C, 700 °C, and 800 °C, respectively). A good trade-off between the OER activity and stability is achieved at 500 and 600 °C due to their nanofibrous core–shell structure (4.2 and 1.4 times higher at *E* = 1.60 V vs RHE as well as 1.3 and 2.4 times higher than the reference material, respectively). However, the synthesis method presented can thus lead to artifacts in the dissolution rate of accompanying metals such as Ni, Cr, Fe, and Al.

These results highlight the potential application of nanofibers in PEM water electrolysis after scaling up electrospinning technology. Moreover, we confirm that the electrocatalytic activity and S number can also be optimized for these special nanofibers by the calcination temperature. IrO_*x*_ calcined at 600 °C can be the primary nanofiber material to lower the Ir content further by applying alternative electrospinning techniques. Catalyst utilization can thus be further enhanced, and the price of acidic water electrolysis can be decreased. These results provide complex insight into the catalytic behavior of novel electrospun nanofibers and pave the way for an alternative morphology to increase the accessibility of catalyst active centers and to enhance catalyst utilization.

## Experimental Section

### Chemicals

Iridium chloride hydrate (IrCl_3_·*x*H_2_O, 99.8% metal basis) and iridium oxide (IrO_*x*_ Premion Alfa Aesar 99.99%, Ir 84.5% min.) were purchased from Thermo Fisher; poly(vinylpyrrolidone) (PVP, *M*_w_ = 1.300.000), isopropyl alcohol (IPA Emsure, ≥99.8%), acetone (ACN Emsure, ≥99.8%), Nafion (1100W dispersion, 5 wt %), and perchloric acid (HClO_4_ Suprapur, 70%) were purchased from Sigma-Aldrich. Glassy carbon plates (GC, 5 × 5 cm^2^, HTW Sigradur G) were supplied by HTW, and potassium hydroxide (KOH, 99.98%) was obtained from Carl Roth. Nitric acid (HNO_3_ Suprapur, 60%) was purchased from Merck. Deionized water (Millipore, 18.2 MΩ cm at 25 °C, TOC < 3 ppb) was used to prepare the aqueous solutions.

### Nanofiber Synthesis

For the calcination study, a total of 1.65 g of IrCl_3_·*x*H_2_O was dissolved in deionized water, and the precursor solution was homogenized in an ultrasonic bath for 15 min. The solution was heated to 80 °C on a hot plate and constantly stirred for 12 h, while PVP was gradually added to reach 15 wt % with respect to the polymer and 3.3 wt % to the precursor salt. The solution was cooled to ambient temperature before the electrospinning process. Electrospinning was performed on a Nanofiber Electrospinning Unit (Kato Tech Co.) with a rotating drum collector and climate chamber. The precursor nanofibers were electrospun in a strong electric field with an applied voltage of *E* = 20 kV. An aluminum foil was placed on the drum collector at a distance of *d* = 10 cm from the needle tip to the collector. The flow rate of the solution was set at 80 μL h^–1^. After electrospinning, the total amount of the nanofiber mat was divided into six fractions and placed in an oven under synthetic air (the flow rate was set at *V̇* = 100 mL min^–1^). Calcination took place at various temperatures (*T* = 400 °C, 500 °C, 600 °C, 700 °C, and 800 °C) for 2 h each. The heating rate was set to 1 K min^–1^. This resulted in an IrO_*x*_ nanofiber mat that could be used directly for ink preparation, as it turns into individual fibers during the subsequent ultrasonication step.^36^

### Ink Preparation

Both IrO_*x*_ nanofibers and the reference IrO_*x*_ Alfa Aesar (AA) were dispersed in a mixture of IPA:H_2_O (7:1). A Nafion ionomer solution was added as a binder to suppress catalyst detachment (ionomer-to-catalyst weight ratio I/C = 4.5:1). The dispersion was homogenized in an ice bath through the application of an ultrasonic horn (Branson Ultrasonics SFX150). The ultrasonication process took 15 min involving the constant repetition of steps in which the sonicator was on for 4 s and then off for 2 s. 0.1 M KOH was then added to the dispersion to adjust to pH = 11 with an HI5521 benchtop meter (Hanna Instruments) after calibration. Before drop-casting, the glassy carbon (GC) plate was polished using the Struers LaboForce-100 polishing machine to obtain a smooth surface and subsequently rinsed with water and ACN. Catalyst spots were then deposited from the as-prepared ink onto the GC surface in a single aliquot of 0.2 μL.^11^ The catalyst loading of a single spot was approximately 10 μg_Ir_ cm^–2^ depending on its size. The latter was determined with a Keyence VK-X250 profilometer.

### Physical Characterization

#### XPS

X-ray photoelectron spectroscopy measurements were carried out on a Quantera II (Physical Electronics) device equipped with a monochromatic Al K_α_-X-ray source (1486.6 eV), which was operated at 15 kV and 25 W. Spectra were acquired over a circular area with a diameter of 100 μm from drop-cast samples on Pt-sputtered Si wafer substrates. Automatic charge neutralization was used for all measurements. The spectra were analyzed using the software Casa XPS (v2.3.24). A Gaussian–Lorentzian function was used to fit the contributions of lattice oxygen, OH-groups, and H_2_O molecules in the O 1s spectra. To deconvolve the Ir 4f peak, we adapted the approach of Freakley et al.^52^ and used asymmetric line shapes with a damped tail to separately fit the contributions of Ir^0^, Ir^+4^, and Ir^+3^ species and a Gaussian–Lorentzian function to fit satellite structures. The parameters used for the deconvolution of all spectra can be found in Tables S1–S3.

#### XRD

All samples were measured as drop-cast films on a silicon sample holder in a Bruker Advance D8 diffractometer (Cu K_α_: λ = 1.5418 Å). Measurements were conducted with grazing incidence in a range of 2θ of 20° to 80° with an increment of 0.02° and a measuring time of 7 s per step where θ is the angle between the outgoing electromagnetic wave and the crystallographic reflecting plane.

#### BET

Nitrogen adsorption analysis was conducted by obtaining physisorption isotherms at 77 K with a Micromeritics TriStar II Plus instrument to determine the specific activity of the IrO_*x*_ catalyst materials. The powder samples were degassed in a glass tube at 250 °C for 12 h. The isotherms were recorded between *p*/*p*^0^ = 0.05 and 0.90 (*p*: gas pressure, *p*^0^: saturation pressure). The evaluation was done by the Brunauer–Emmett–Teller method (BET) between *p*/*p*^0^ = 0.20 and 0.60.^67^

#### SEM

The morphology of the fibers was examined before and after calcination using a Tescan Vega 3 (MIRA 3) scanning electron microscope (SEM) operated with a primary electron energy of 20 keV coupled with an EDXS (EDAX Octane Elect EDS detector). All samples were immobilized on a sample holder stub with double-sided adhesive carbon tape. Before calcination, the samples were sputter-coated with gold prior to imaging.

#### TEM

High-angle annular dark-field scanning transmission electron microscopy (HAADF-STEM) and selected area electron diffraction (SAED) were performed using a Thermo Fisher Scientific Talos F200i instrument operated at an acceleration voltage of 200 kV, a beam current of 40 pA, and a convergence angle of 10 mrad. The microscope was equipped with two Bruker XFlash 6 | 100 detectors for energy-dispersive X-ray spectroscopy (EDXS) mapping. For SAED, an aperture with a diameter of 200 μm and a camera length of 1.1 m was used. Patterns were acquired using a Ceta-S CMOS camera with 4k × 4k pixels. The radial profile of SAED patterns was obtained by polar transformation around the radial center of the patterns and distortion-corrected up to the fourth order using a Python code introduced elsewhere.^68^ The background signal was subtracted based on a power law intensity decay, as described in the Supporting Information (SI).

### Electrochemical Characterization and Stability Measurements (SFC-ICP-MS)

All electrochemical measurements were conducted in a scanning flow cell (SFC) designed in-house, which has been described elsewhere.^69^ In order to obtain online data about elemental dissolution, the outlet channel of the SFC was coupled to an inductively coupled plasma mass spectrometer (Agilent 7900 ICP-MS, Agilent). 0.1 M HClO_4_ was prepared and purged with Ar prior to each measurement. The electrolyte was pumped through the SFC from a reservoir by the peristaltic pump of the ICP-MS and mixed with the proper internal standard solution (ratio 2:1) before reaching the mass spectrometer. The device was calibrated on a daily basis with standard solutions of 0.5, 1, and 5 ppb. The flow rate was kept at a constant level of 341 μL min^–1^. The opening of the SFC was 0.036 cm^2^. We measured ^193^Ir (ISTD: 3 μg l^–1 187^Re), ^27^Al (ISTD: 10 μg l^–1 45^Sc). For ^60^Ni, ^56^Fe, and ^52^Cr, we used 50 μg l^–1 72^Ge as the ISTD.

All electrochemical measurements were conducted with a potentiostat/galvanostat (Reference 620, Gamry Instruments). As for the counter and reference electrodes, a reversible hydrogen electrode (Hydroflex Mini, Gaskatel) and a GC rod (*d* = 1.6 mm, HTW Sigradur G) were selected and placed into the inlet and outlet channels of the SFC, respectively. The standard potential of the RHE was regularly determined in a conventional benchmark cell against Pt in 0.1 M HClO_4_ solution and continuous H_2_ bubbling before the experiments. In this study, all potentials are presented vs RHE at 25 °C. As mentioned above, drop-cast IrO_*x*_ catalyst spots on the backing electrode were tested as the working electrode. Potentiostatic electrochemical impedance spectroscopy (PEIS) measurements were used to assess the impedance of the electrolyte. Impedance spectra were recorded in the frequency range of 100 kHz–1 kHz using a sinusoidal excitation signal with an amplitude of ±10 mV vs the previously measured OCP. Linear sweep voltammetry (LSV) was conducted from 0.80 or 1.10 V to 1.70 V vs RHE to gain first-hand information on catalyst activity. Cyclic voltammetry (CV) was also applied between 0.05 and 1.30 V vs RHE with a scan rate of 20 mV s^–1^. At least three repeated measurements were performed for each catalyst material. Data evaluation and visualization of electrochemistry results took place via integrated database communication to bring about accelerated materials research.^70^

## Supporting Information Available

SEM data, TGA results, STEM and distortion-corrected SAED profiles, summary of XPS data in tables, supporting data of electrochemical characterization (CVs and electrocatalytic activity during galvanostatic hold protocol), dissolution study of coexisting metals (PDF)
