# Supplementary material for: Electrospun Iridium-Based Nanofiber Catalysts for Oxygen Evolution Reaction: Influence of Calcination on Activity–Stability Relation
Source: ACS Appl Mater Interfaces. 2024 Sep 18;16(39):52179–90. doi: 10.1021/acsami.4c07831 (PMC11450683; doi:10.1021/acsami.4c07831)
Supplement: Supplementary file 1 — am4c07831_si_001.pdf [file am4c07831_si_001.pdf]

Supporting Information

Electrospun Iridium-Based Nanofiber Catalysts for Oxygen Evolution Reaction: Influence of Calcination on Activity-Stability Relation

Miklós Márton Kovács,*,†,‡ Birk Fritsch,† Leopold Lahn,†,¶,§ Julien Bachmann,‖ Olga Kasian,†,¶,§ Karl J. J. Mayrhofer,†,‡ Andreas Hutzler,† and Dominik Dworschak*,†

†*Forschungszentrum Jülich GmbH, Helmholtz Institute Erlangen-Nürnberg for Renewable Energy (IEK-11), 91058 Erlangen, Germany*

‡*Friedrich-Alexander-Universität Erlangen-Nürnberg, Department of Chemical and Biological Engineering, 91058 Erlangen, Germany*

¶*Helmholtz-Zentrum Berlin für Materialien und Energie GmbH, Dynamic Electrocatalytic Interfaces, Hahn-Meitner-Platz 1, 14109 Berlin*

§*Friedrich-Alexander-Universität Erlangen-Nürnberg, Department of Materials Science and Engineering, 91058 Erlangen, Germany*

‖*Friedrich-Alexander-Universität Erlangen-Nürnberg, Chemistry of Thin Film Materials, IZNF, 91058 Erlangen, Germany*

E-mail: m.kovacs@fz-juelich.de; d.dworschak@fz-juelich.de

Physical characterization

SEM

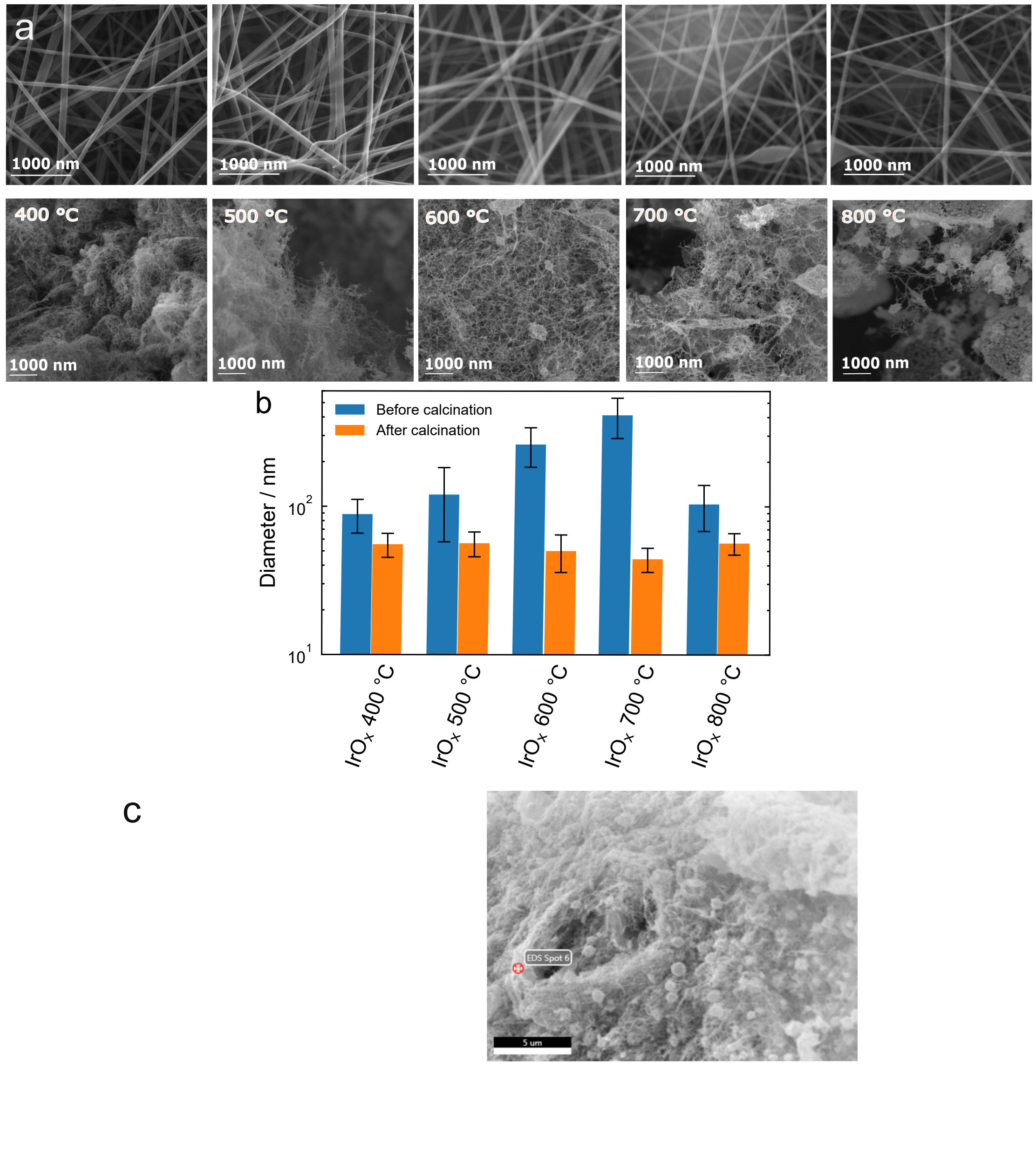

Figure S1: a) SEM images of Ir-based samples before and after calcination ($IrCl_3$/PVP nanofibers (NFs) and IrO_x NFs, respectively). b) Fiber diameter of Ir-based NFs and c) EDXS study of IrO_x 600 °C where the chemical composition of the fibers was investigated. It illustrates the relative proportion of the existing elements at a singular location within the IrO_x 600 °C specimen, as demonstrated in the inset. Besides oxygen and iridium, a sharp peak of aluminum can be observed deriving from the alumina crucible in which calcination is conducted.

TGA

The thermal stability of the as-spun $IrCl_3$/PVP nanofibers and the calcined IrO_x samples was analyzed by thermogravimetric analysis (TGA, TGA8000, Perkin Elmer) between 30 and 1000 °C at a heating rate of 10 K min^{-1}. In order to observe the oxidation process, the analysis took place under synthetic air atmosphere. The flow rate was selected 30 ml min^{-1}.

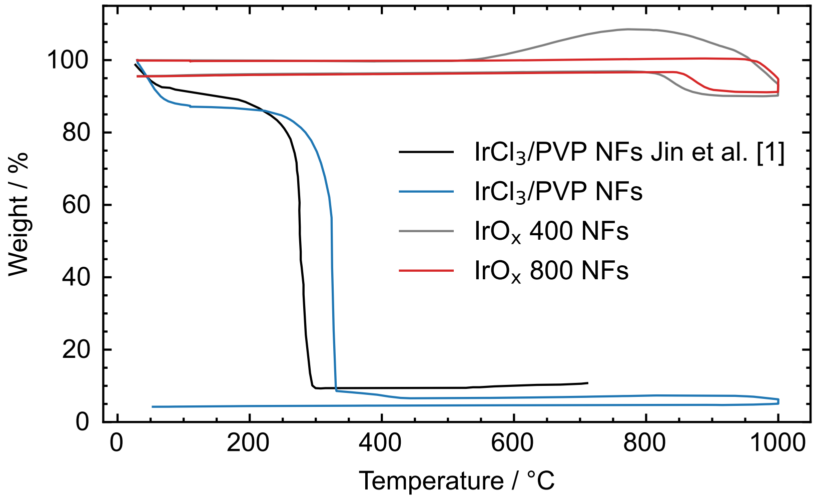

Figure S2: Thermogravimetrical analysis of as-spun $IrCl_3$/PVP nanofibers and as-calcined IrO_x 400 °C and 800 °C. Mass losses during TGA of a previous report are reproduced and adapted with permission from Ref.[1]

STEM

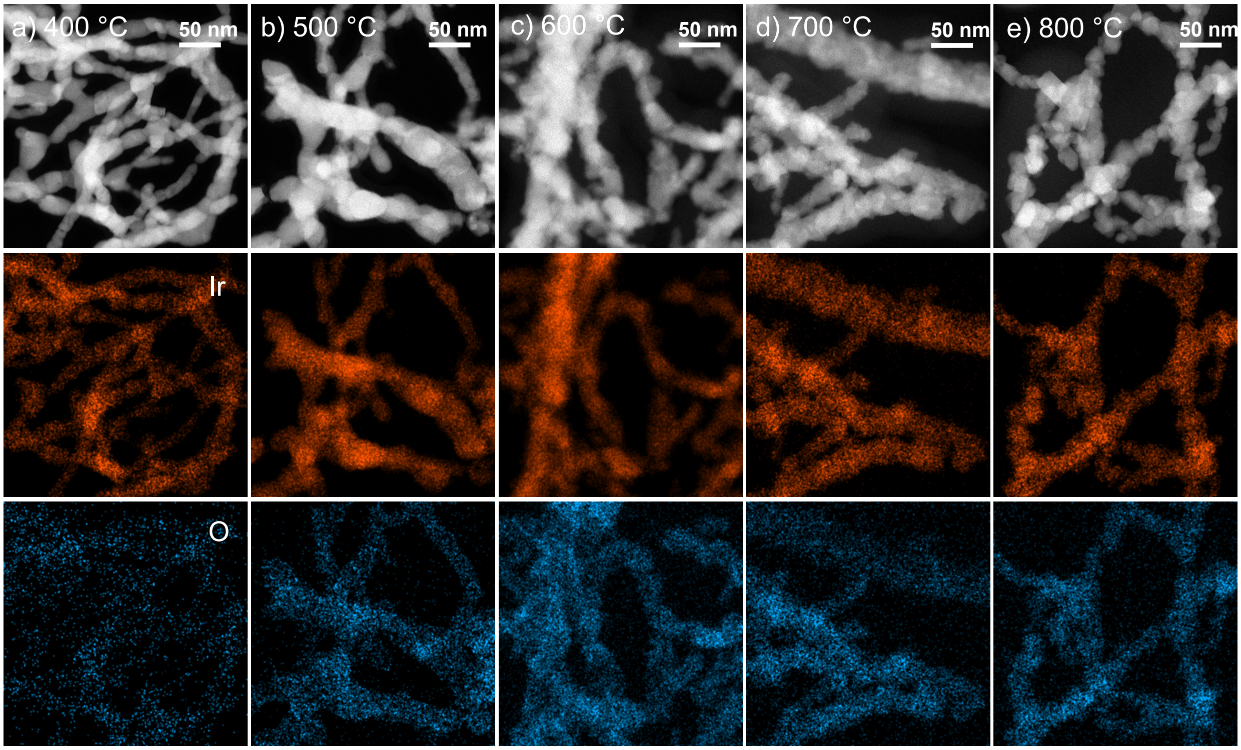

Figure S3: HAADF-STEM and STEM-EDXS spectrum images of Ir and O distribution for a) nanofibrous IrO_x catalyst materials calcined at a) 400 °C, b) 500 °C, c) 600 °C , d) 700 °C, and e) 800 °C.

Distortion correction of SAED profiles

Selected area electron diffraction (SAED) patterns were analyzed using a procedure elucidated elsewhere.[2] First, the precise position of the zero beam was determined by circular Hough transform and ellipse fitting. After subsequent polar transform, distortions up to fourth order were corrected using:

$$R(\phi) = R_0 \sum_{\mathrm{k}=2}^{4} \frac{1-\eta_{\mathrm{k}}^2}{\sqrt{1+\eta_{\mathrm{k}}^2 - 2\eta_{\mathrm{k}} \cos(\mathrm{k}(\phi + \omega_{\mathrm{k}}))}} \tag{1}$$

Here, R is the diffraction ring radius as a function of the polar angle ϕ. R_0 describes its undistorted value. Moreover, η_{k} describes the distortion strength of the kth order. The orientation of η_{k} with respect to its major axis is given by ω_{k}. The pixel intensity was used

as a weigthing factor for least-squares fitting. Distortions were corrected on the 220 fcc ring for the samples calcined at 400 °C, 500 °C, and 600 °C. For the specimen calcined at 700 °C and 800 °C, the rutile 211 ring was chosen. Figure S4 displays this exemplarily for the 700 °C sample.

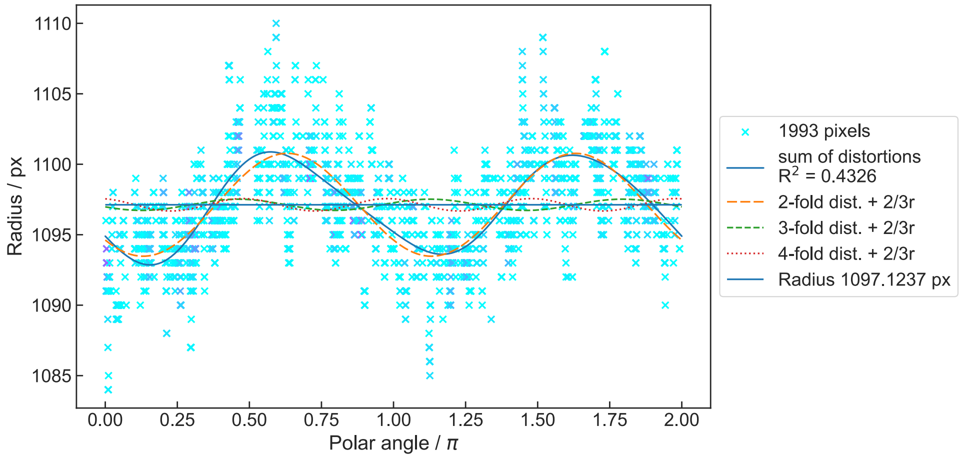

Figure S4: Exemplary distortion fit on the 700 °C sample. The darkness of the data points correspond to their intensity.

Next, the corrected data was integrated along ϕ, yielding distortion-corrected radial profiles. To subtract the contribution of the zero beam, a power law was fitted to the data in-between the Bragg peaks (highlighted regions in Figure S5). Normalization to [0, 1] yields the data presented in Figure 4f of the main manuscript.

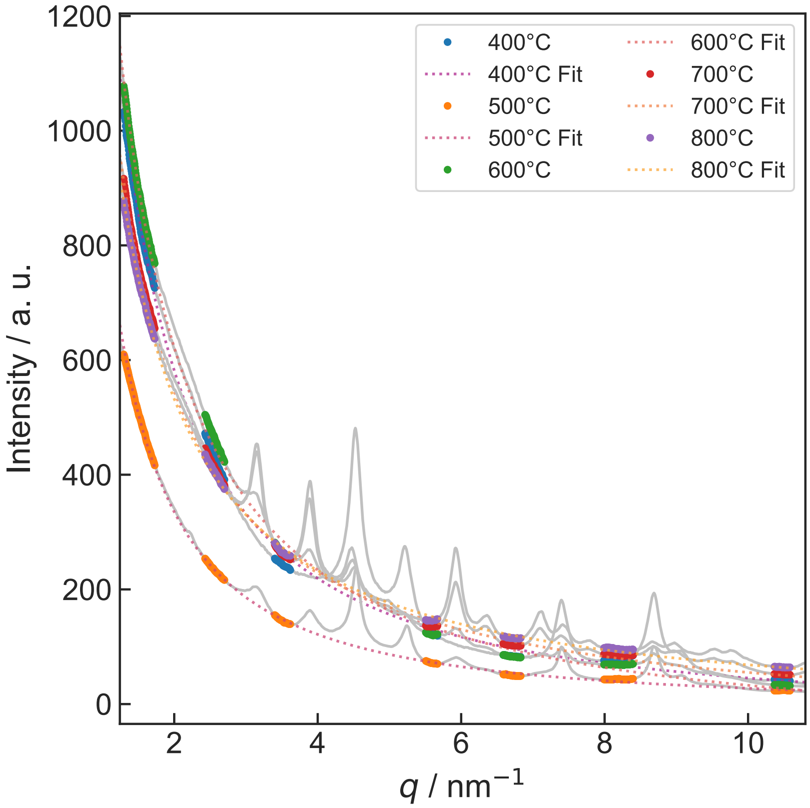

Figure S5: Power law fitting to distortion-corrected radial SAED profiles. The colored regions denote the data used for fitting.

XPS

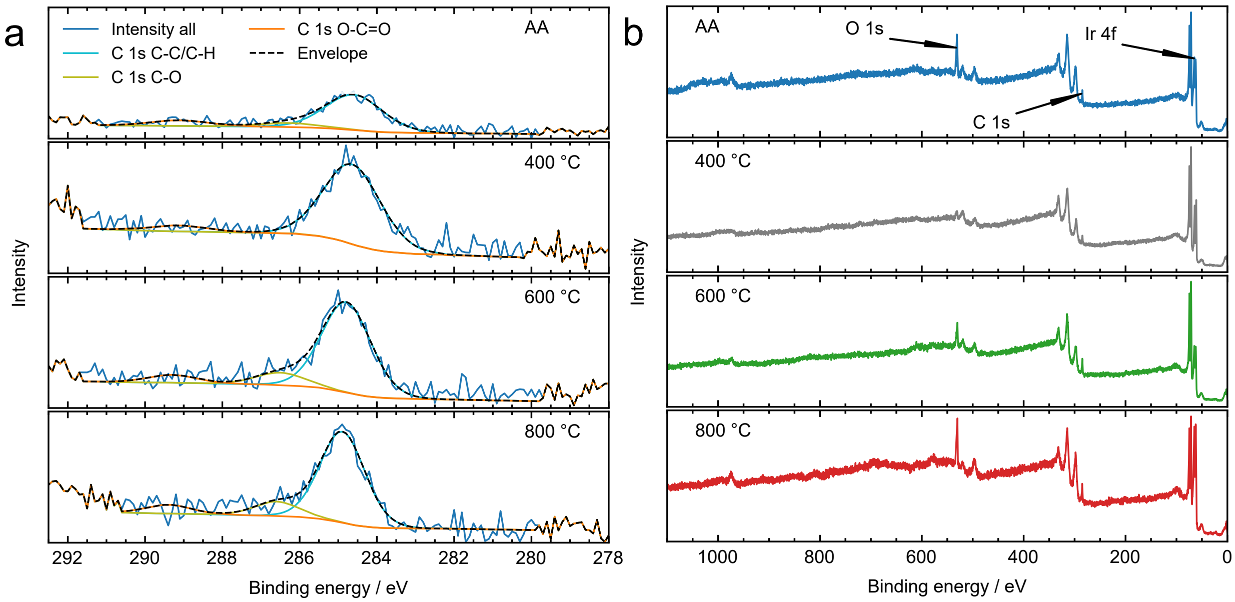

Figure S6: XPS analysis of a) C 1s spectra and b) survey spectra of Ir 4f and O 1s of the benchmark material IrO_x Alfa Aesar (AA) and the nanofibrous IrO_x catalyst materials calcined at various temperatures of T = 400 °C, 600 °C, and 800 °C.

Table S1: Fitting parameters used for deconvolution of the Ir 4f peaks in all samples.

sample	peak	contribution	binding energy [eV]	FWHM [eV]	area [%]	CasaXPS lineshape
**IrO_x**	Ir $4f_{7/2}$	Ir^0	60.9	0.9	1.6	LF(0.6,1,150,300)
**Alfa Aesar**	Ir $4f_{5/2}$	Ir^0	63.9	0.9	1.2	LF(0.6,1,150,300)
	Ir $4f_{7/2}$	Ir^{+4}	61.7	0.9	37.6	LF(0.3,1,65,150)
	Ir $4f_{5/2}$	Ir^{+4}	64.7	0.9	28.2	LF(0.3,1,65,150)
	Ir $4f_{7/2}$	Ir^{+4} satellite	63.0	2.1	4.9	GL(50)
	Ir $4f_{5/2}$	Ir^{+4} satellite	66.0	2.1	3.7	GL(50)
	Ir $4f_{7/2}$	Ir^{+3}	62.3	0.9	13.1	LF(0.3,1,65,150)
	Ir $4f_{5/2}$	Ir^{+3}	65.3	0.9	9.5	LF(0.3,1,65,150)
**IrO_x**	Ir $4f_{7/2}$	Ir^0	61.0	0.8	45.0	LF(0.6,1,150,300)
**400 °C**	Ir $4f_{5/2}$	Ir^0	64.0	0.8	33.7	LF(0.6,1,150,300)
	Ir $4f_{7/2}$	Ir^{+4}	61.8	0.8	12.2	LF(0.3,1,65,150)
	Ir $4f_{5/2}$	Ir^{+4}	64.8	0.8	9.1	LF(0.3,1,65,150)
**IrO_x**	Ir $4f_{7/2}$	Ir^0	61.0	0.9	8.5	LF(0.6,1,150,300)
**600 °C**	Ir $4f_{5/2}$	Ir^0	64.0	0.9	6.3	LF(0.6,1,150,300)
	Ir $4f_{7/2}$	Ir^{+4}	61.8	0.9	46.8	LF(0.3,1,65,150)
	Ir $4f_{5/2}$	Ir^{+4}	64.8	0.9	35.1	LF(0.3,1,65,150)
	Ir $4f_{7/2}$	Ir^{+4} satellite	63.1	1.6	1.9	GL(50)
	Ir $4f_{5/2}$	Ir^{+4} satellite	66.1	1.6	1.4	GL(50)
**IrO_x**	Ir $4f_{7/2}$	Ir^{+4}	61.7	0.9	53.7	LF(0.3,1,65,150)
**800 °C**	Ir $4f_{5/2}$	Ir^{+4}	64.7	0.9	40.3	LF(0.3,1,65,150)
	Ir $4f_{7/2}$	Ir^{+4} satellite	63.0	2.2	3.4	GL(50)
	Ir $4f_{5/2}$	Ir^{+4} satellite	66.0	2.2	2.6	GL(50)

Table S2: Fitting parameters used for deconvolution of the O 1s peaks in all samples.

sample	peak	contribution	binding energy [eV]	FWHM [eV]	area [%]	CasaXPS lineshape
**IrO_x**	O 1s	oxide	530.1	0.9	5.0	GL(50)
**Alfa Aesar**		OH	531.2	2.3	80.5	GL(50)
		H_2O	533.3	2.3	14.5	GL(50)
**IrO_x**	O 1s	oxide	530.0	0.8	11.6	GL(50)
**400 °C**		OH	531.1	2.2	62.3	GL(50)
		H_2O	533.2	2.2	26.1	GL(50)
**IrO_x**	O 1s	oxide	530.0	0.9	22.8	GL(50)
**600 °C**		OH	531.1	2.2	60.1	GL(50)
		H_2O	533.2	2.2	17.1	GL(50)
**IrO_x**	O 1s	oxide	530.0	0.8	27.0	GL(50)
**800 °C**		OH	531.1	2.2	61.4	GL(50)
		H_2O	533.2	2.2	11.6	GL(50)

Table S3: Concentration of the contributions of metallic Ir (Ir^0), hydrous Ir oxides (Ir^{+3}) and stoichiometric Ir oxides (Ir^{+4}) in all samples, derived from the deconvolution of Ir 4f peaks.

sample	Ir^0 [at. %]	Ir^{+3} [at. %]	Ir^{+4} [at. %]
**IrO_x Alfa Aesar**	3.0	25.0	72.0
**IrO_x 400 °C**	78.7	0	21.3
**IrO_x 600 °C**	15.3	0	84.7
**IrO_x 800 °C**	0	0	100

Electrochemical characterization

Cyclic voltammetry and electrocatalytic activity

The conditioning of the spot surface is executed by means of cyclic voltammetry (CV) between +0.05 V and +1.30 V *vs.* RHE to observe its potential influence on the electrocatalytic activity determined by LSVs. The voltammetry measurement comprises three cycles on the catalyst spots and are summarized in Figure S7.a taking IrO_x NFs calcined at 400 °C, 500 °C, 700 °C, and 800 °C as examples. Each material is investigated once, and the scan rate was selected as being 20 mV s^{-1}. The impact of cyclic voltammetry on the OER activity of all samples including the reference material is summarized in Table S4.

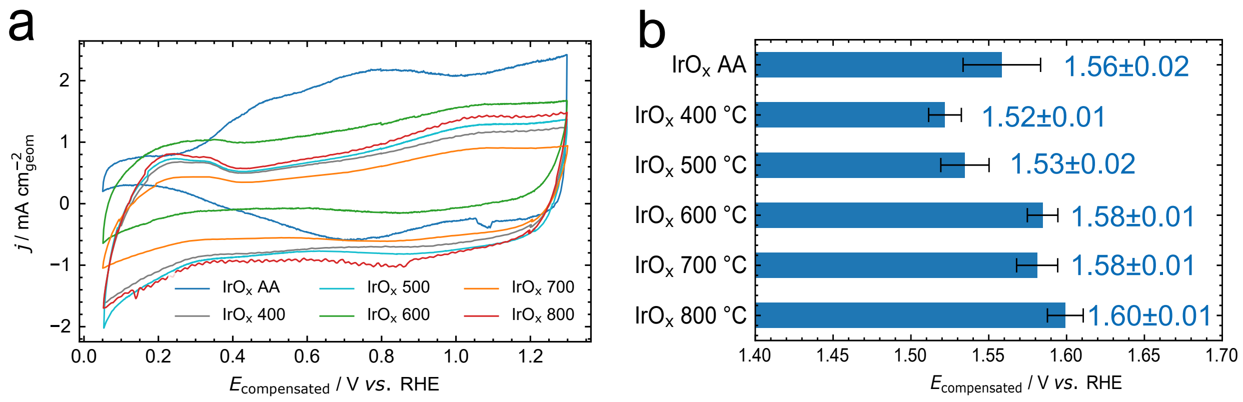

Figure S7: a) Cyclic voltammetry (CV) of the last cycle of the reference material IrO_x AA and IrO_x NFs calcined at 400 °C, 500 °C, 600 °C, 700 °C, and 800 °C. CVs were conducted between E = +0.05 V and +1.30 V *vs.* RHE, the scan rate was selected 20 mV s^{-1}. b) Potential (iR corrected) obtained during the galvanostatic hold of 1 mA cm^{-2} of IrO_x NFs after calcination at T = 400 °C, 500 °C, 600 °C, 700 °C, and 800 °C as well as the reference in 0.1 M $HClO_4$. The duration of the galvanostatic step is 600 s. We notify that the applied current density is not sufficiently high to guarantee that the generated potential arises solely from the oxygen evolution as it may induce oxidation reactions (e.g. oxide transition).

Table S4: Impact of CV on the OER activity. Current density of IrO_x calcined at 400 °C, 500 °C, 600 °C, 700 °C, and 800 °C as well as the reference material IrO_x Alfa Aesar (AA) is summarized at 1.55 V and 1.60 V *vs.* RHE with (w/) and without (w/o) CVs.

sample	$\boldsymbol{j}$ [mA cm^{-2}] at 1.55 V *vs.* RHE w/o CV	$\boldsymbol{j}$ [mA cm^{-2}] at 1.55 V *vs.* RHE w/ CV	$\boldsymbol{j}$ [mA cm^{-2}] at 1.60 V *vs.* RHE w/o CV	$\boldsymbol{j}$ [mA cm^{-2}] at 1.60 V *vs.* RHE w/ CV
**IrO_x AA**	1.22±0.39	$7.88 \cdot 10^{-1}$	3.71±1.54	2.77
**IrO_x 400 °C**	6.13±2.85	4.16	$(1.46 \pm 0.85) \cdot 10^{1}$	8.01
**IrO_x 500 °C**	3.73±2.10	7.33	$(1.55 \pm 0.43) \cdot 10^{1}$	$2.52 \cdot 10^{1}$
**IrO_x 600 °C**	$(9.80 \pm 2.98) \cdot 10^{-1}$	$8.90 \cdot 10^{-1}$	5.33±1.69	5.53
**IrO_x 700 °C**	$(8.37 \pm 1.72) \cdot 10^{-1}$	2.08	4.86±1.34	4.49
**IrO_x 800 °C**	$(4.08 \pm 0.45) \cdot 10^{-1}$	3.07	2.62±0.49	4.78

Table S5: Comparison of the mass-specific (*MA*) and area-specific (*SA*) activities of IrO_x AA and nanofibrous IrO_x catalyst materials calcined at various temperatures between 400 °C and 800 °C for the OER at 1.55 V and 1.60 V *vs.* RHE determined from the LSV (1.10 → 1.70 V) at 20 mV s^{-1} for thin film samples.

**Sample**	***ECSA***	**norm. *ECSA***	***MA* at 1.55 V**	***MA* at 1.60 V**	***SA* at 1.55 V**	***SA* at 1.60 V**	**BET**
	cm^2	$m^2\,g^{-1}_{Ir}$	$A\,mg^{-1}_{Ir}$	$A\,mg^{-1}_{Ir}$	$mA\,cm^{-2}_{BET}$	$mA\,cm^{-2}_{BET}$	$m^2\,g^{-1}_{Ir}$
**AA**	2.36	1431.90	$(1.22\pm0.39)\cdot10^{-1}$	$(3.71\pm1.54)\cdot10^{-1}$	0.41	1.25	29.81
**400**	1.63	985.28	$(6.13\pm2.85)\cdot10^{-1}$	1.46 ± 0.85	1.83	4.36	33.57
**500**	1.50	905.89	$(3.73\pm2.10)\cdot10^{-1}$	1.55 ± 0.43	–	–	–
**600**	1.87	1133.96	$(9.80\pm2.98)\cdot10^{-2}$	$(5.33\pm1.69)\cdot10^{-1}$	0.52	2.81	18.96
**700**	1.54	931.99	$(8.37\pm1.72)\cdot10^{-2}$	$(4.86\pm1.34)\cdot10^{-1}$	–	–	–
**800**	1.60	971.84	$(4.08\pm0.45)\cdot10^{-2}$	$(2.62\pm0.49)\cdot10^{-1}$	0.35	2.26	11.62

The normalized *ECSA* values are an order of magnitude higher than for platinum[3] which originates from the ECSA determination technique applied.[4,5] The loading of each spot is L_{Ir} = 10.00 µg cm^{-2} and the mass of iridium amounts to 0.165 µg in each spot.

Dissolution study - stability measurements

With regard to the dissolution behavior, we observe sharp peaks of Ni, Cr, Fe, and Al, emerging independently of the steps of the characterization protocol. Such phenomena can stem from bubble transfer from the purge vial to the SFC and appear to be independent from the electrocatalytic protocol applied. As for Al, the constant dissolution might be the result of the presence of Al throughout the nanofibers due to physical adsorption during the calcination process. We obtain evidence of the presence of Al by SEM-EDXS in as-calcined samples (Figure S1.c). This may explain the Al dissolution rates being two orders of magnitude higher than that of the other metals detected. In conclusion, we state that a direct dependence of the dissolution of ^{60}Ni, ^{52}Cr, and ^{56}Fe on the applied electrochemical protocol can not be observed. Regarding ^{27}Al, we conducted control experiments to find out the source of its increased dissolution. First, ^{27}Al interferes with the $^{54}Cr^{++}$ and $^{54}Fe^{++}$ isotopes in the argon plasma of the ICP-MS, i.e. counts of these metals may be detected as ^{27}Al counts. Second, we investigated the dissolution of these metals on the pure background (glassy carbon substrate). Hereby, we detected high level of dissolution of Al during the electrochemical protocol which was comparable to the level of the catalyst spots. The impact of Al on the Ir dissolution will be investigated in our next study.

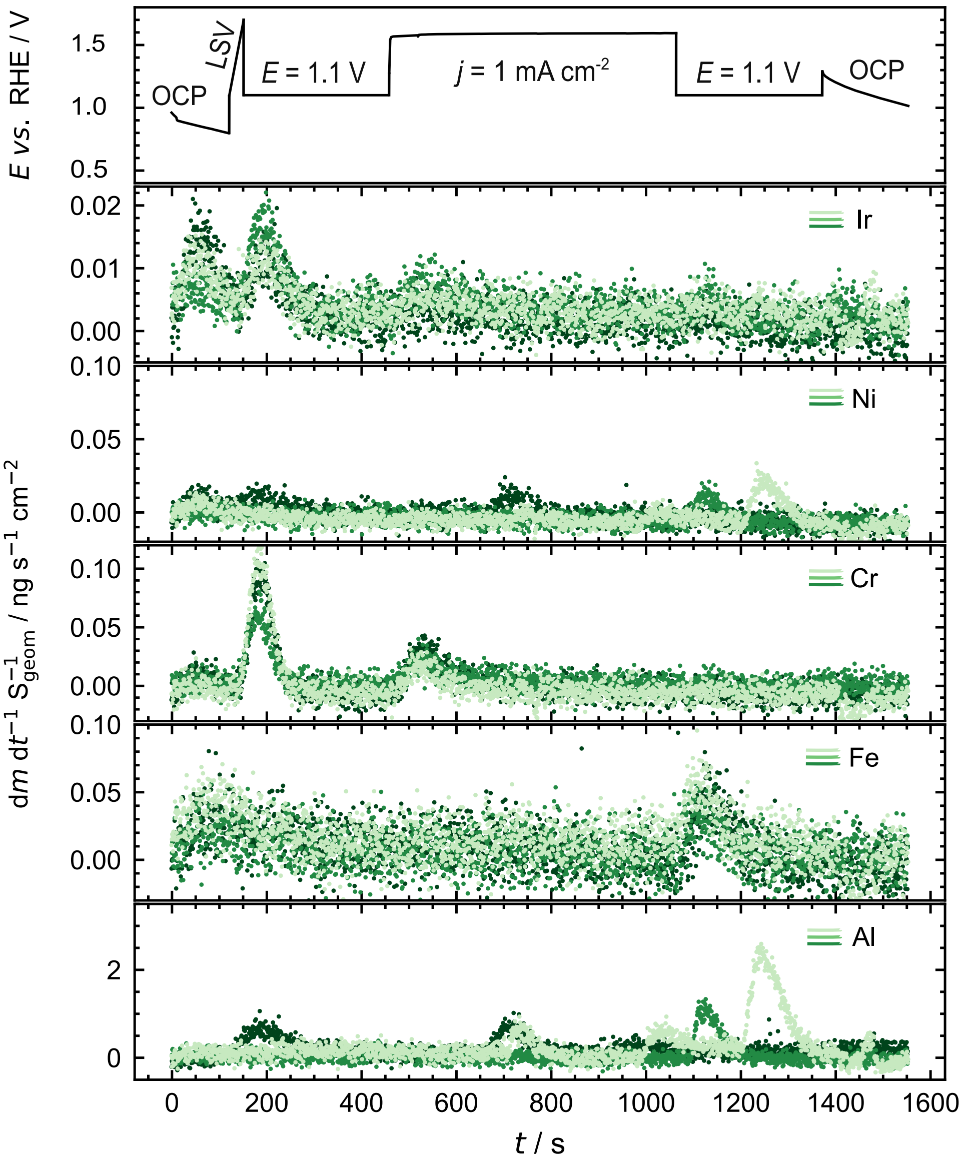

Figure S8: Overall electrochemical characterization protocol (top axis) as well as the dissolution rates of Ir, Ni, Cr, Fe, and Al for the example of IrO_x NFs calcined at 600 °C measured by SFC-ICP-MS in 0.1 M $HClO_4$. Three separate catalyst spots are analyzed at each temperature as depicted by color shading. The characterization protocol consisted of 1. OCP, 2. LSV between E = 1.10 V and 1.70 V, 3. potentiostatic hold at E = 1.10 V, 4. galvanostatic hold at j = 1 mA cm^{-2}, 5. potentiostatic hold at E = 1.10 V, 6. OCP and finally 7. EIS to determine the impedance of the electrolyte for iR correction. All potentials are defined *vs.* RHE at 25 °C.

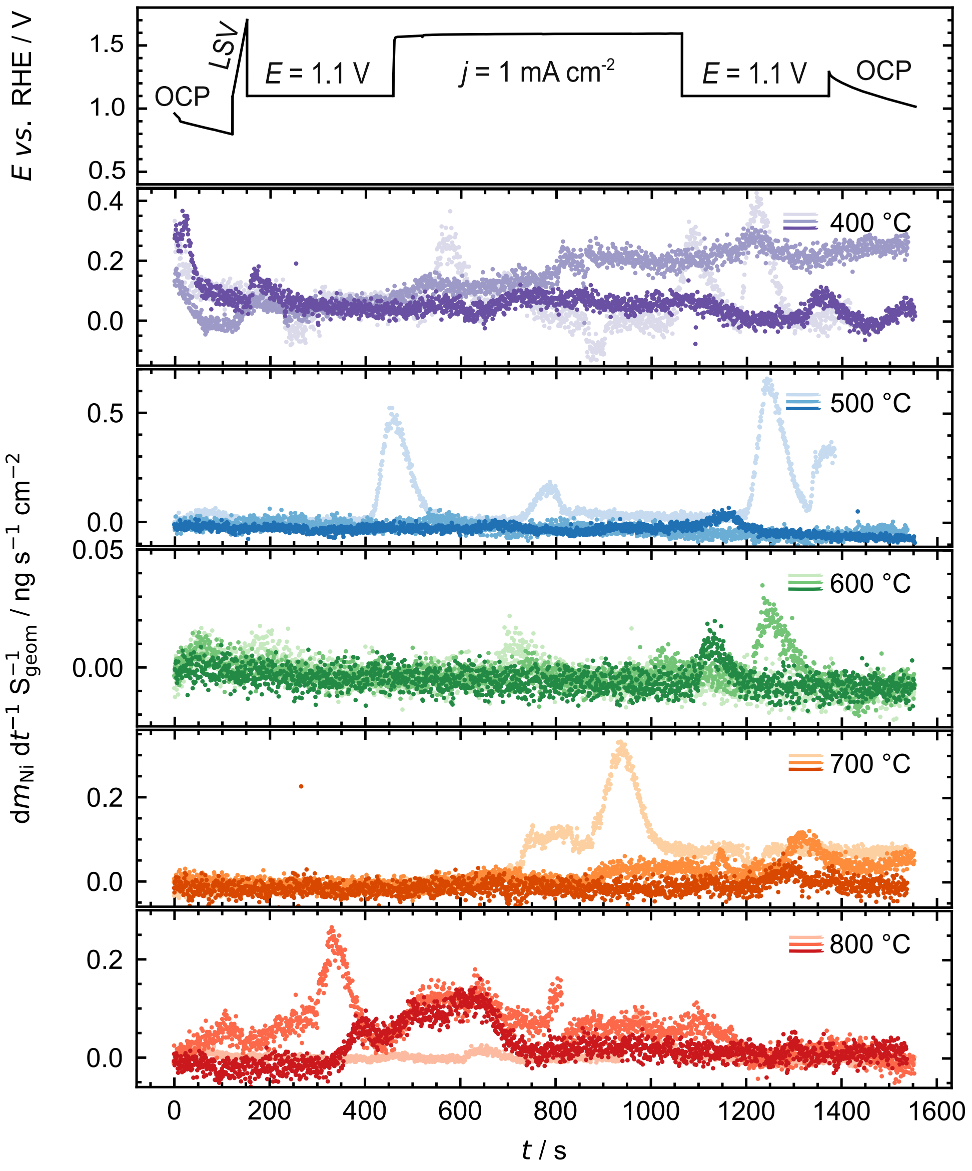

Figure S9: Dissolution rates of Ni in IrO_x NFs during the electrochemical protocol (top axis). Three separate catalyst spots are analyzed at each temperature as depicted by color shading. The potentials are defined *vs.* RHE at 25 °C.

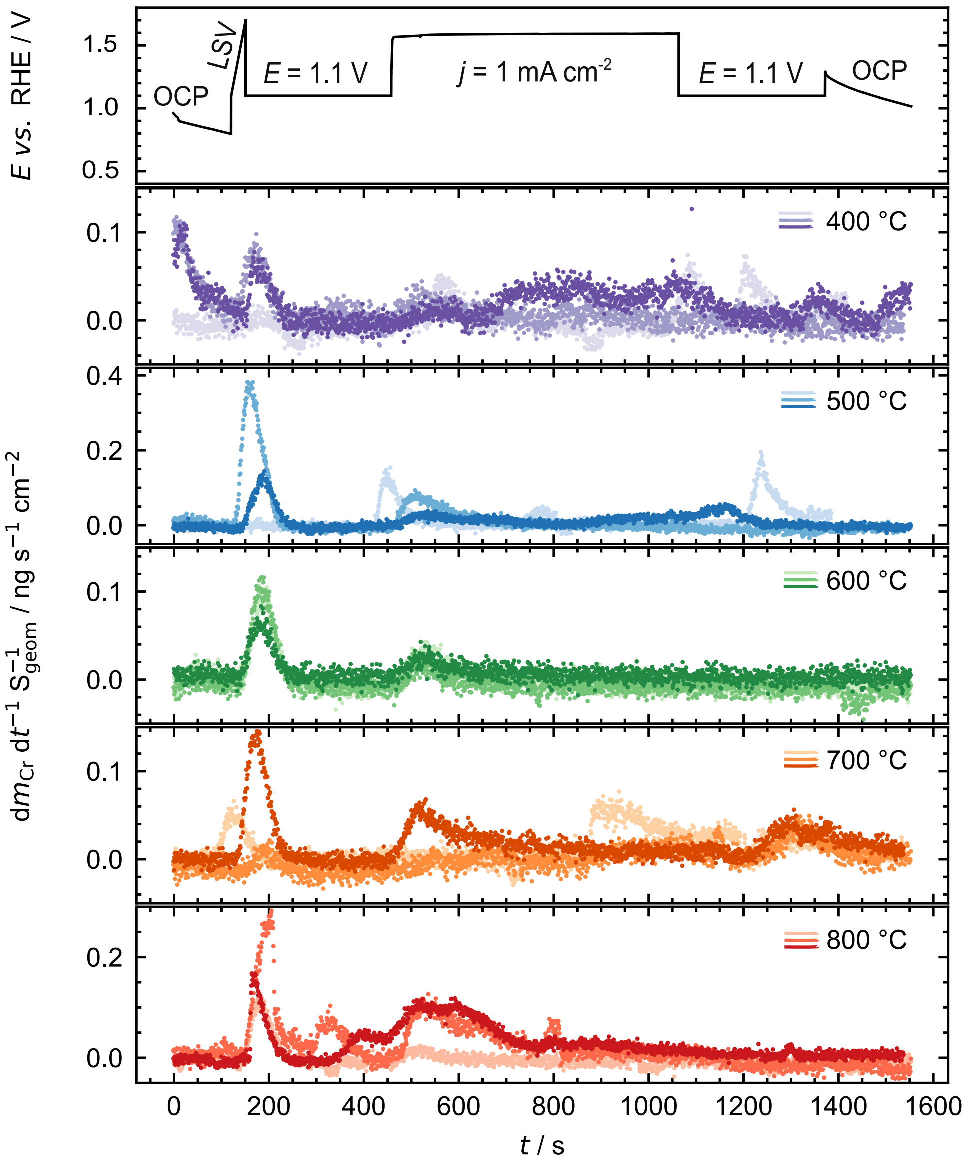

Figure S10: Dissolution rates of Cr in IrO_x NFs during the electrochemical protocol (top axis). Three separate catalyst spots are analyzed at each temperature as depicted by color shading. The potentials are defined *vs.* RHE at 25 °C.

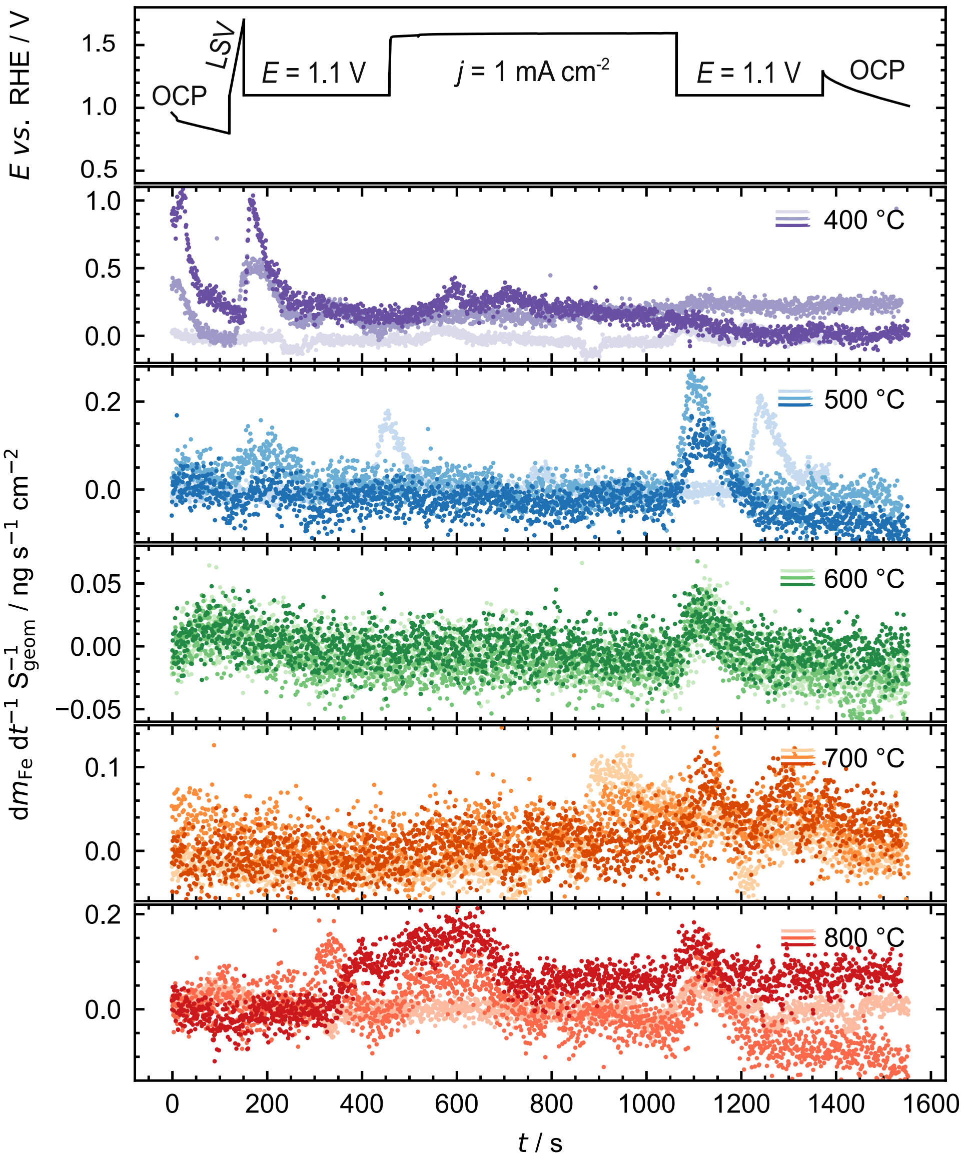

Figure S11: Dissolution rates of Fe in IrO_x NFs during the electrochemical protocol (top axis). Three separate catalyst spots are analyzed at each temperature as depicted by color shading. The potentials are defined *vs.* RHE at 25 °C.

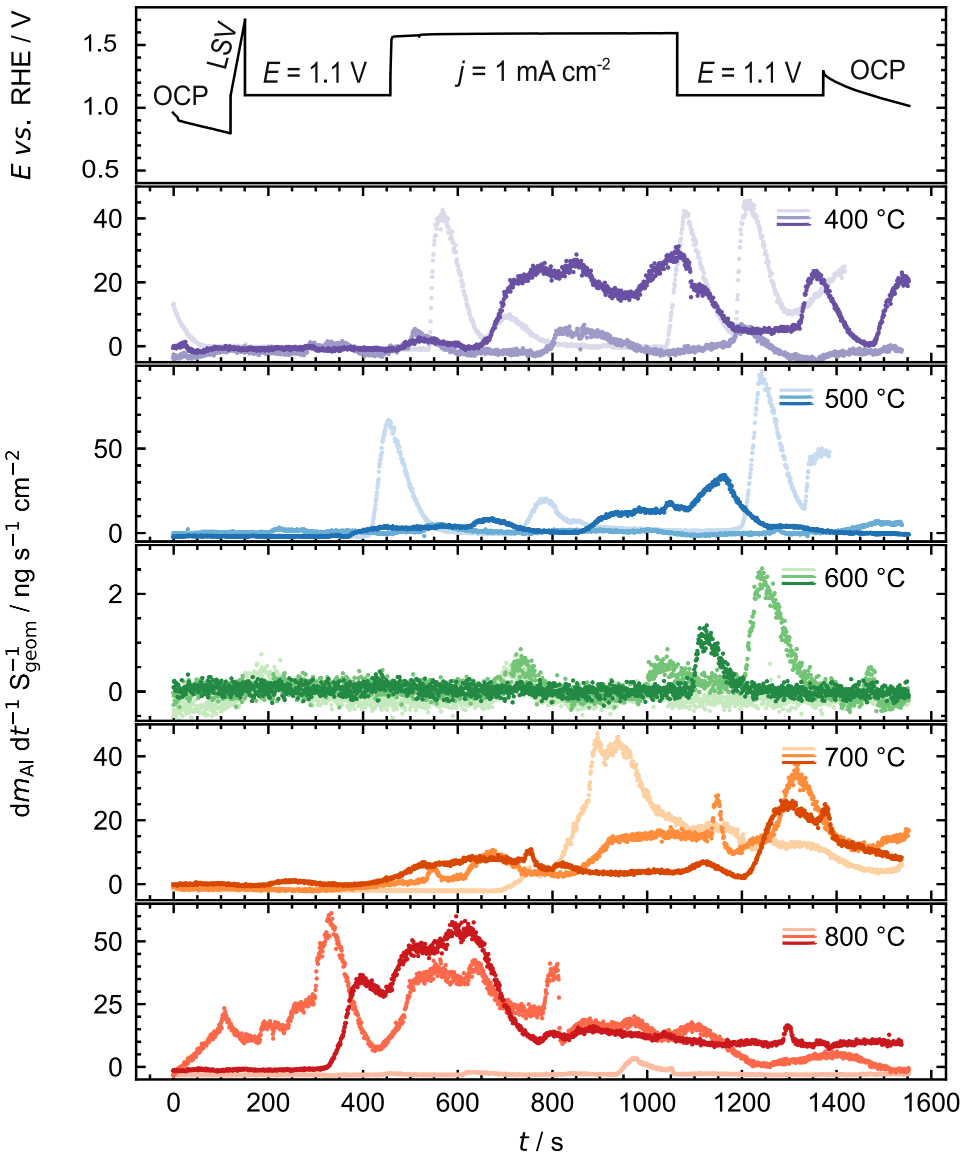

Figure S12: Dissolution rates of Al in IrO_x NFs during the electrochemical protocol (top axis). Three separate catalyst spots are analyzed at each temperature as depicted by color shading. The potentials are defined *vs.* RHE at 25 °C.

References

(1) Jin, D.; Kang, J.; Prabhakaran, S.; Lee, Y.; Kim, M. H.; Kim, D. H.; Lee, C. Chromium-rich Cr x Ir 1− x O 2 wire-in-tube alloys for boosted water oxidation with long standing electrocatalytic activity. *Journal of Materials Chemistry A* **2022**, *10*, 13803–13813.

(2) Birk Fritsch; Mingjian Wu; Andreas Hutzler; Dan Zhou; Ronald Spruit; Lilian Vogl; Johannes Will; H. Hugo Pérez Garza; Martin März; Michael P.M. Jank; Erdmann Spiecker Sub-Kelvin thermometry for evaluating the local temperature stability within in situ TEM gas cells. *Ultramicroscopy* **2022**, *235*, 113494.

(3) Garsany, Y.; Baturina, O. A.; Swider-Lyons, K. E.; Kocha, S. S. Experimental Methods for Quantifying the Activity of Platinum Electrocatalysts for the Oxygen Reduction Reaction. *Analytical Chemistry* **2010**, *82*, 6321–6328.

(4) Reier, T.; Pawolek, Z.; Cherevko, S.; Bruns, M.; Jones, T.; Teschner, D.; Selve, S.; Bergmann, A.; Nong, H. N.; Schlögl, R.; Mayrhofer, K. J. J.; Strasser, P. Molecular Insight in Structure and Activity of Highly Efficient, Low-Ir Ir-Ni Oxide Catalysts for Electrochemical Water Splitting (OER). *Journal of the American Chemical Society* **2015**, *137*, 13031–13040.

(5) Zlatar, M. et al. Standardizing OER Electrocatalyst Benchmarking in Aqueous Electrolytes: Comprehensive Guidelines for Accelerated Stress Tests and Backing Electrodes. *ACS Catalysis* **2023**, *13*, 15375–15392.
